# Dynamic Drusen Remodelling in Participants of the Nutritional AMD Treatment-2 (NAT-2) Randomized Trial

**DOI:** 10.1371/journal.pone.0149219

**Published:** 2016-02-22

**Authors:** Giuseppe Querques, Bénédicte M. J. Merle, Nicole M. Pumariega, Pascale Benlian, Cécile Delcourt, Alain Zourdani, Heather B. Leisy, Michele D. Lee, R. Theodore Smith, Eric H. Souied

**Affiliations:** 1 Department of Ophthalmology - Hôpital intercommunal de Créteil – Université Paris Est Créteil, Créteil, France; 2 Department of Ophthalmology, New York University, New York, New York, United States of America; 3 APHP - Hôpital Saint Antoine - biochemistry and molecular biology department - F-75012 Paris; Université Lille 2, INSERM UMRS 1011, Lille, France; 4 INSERM, Centre INSERM U897-Epidemiologie-Biostatistique, Bordeaux, France; 5 Univ. Bordeaux, ISPED, Bordeaux, France; University of Utah, UNITED STATES

## Abstract

**Purpose:**

To evaluate the dynamic remodeling of drusen in subjects with unilateral neovascular age-related macular degeneration (AMD) receiving a three-year course of oral docosahexaenoic acid (DHA) or placebo.

**Setting:**

Institutional setting.

**Methods:**

Three hundred subjects with age-related maculopathy and neovascular AMD in the fellow eye were randomly assigned to receive either 840 mg/day DHA or placebo for 3 years. Main outcome measures of this post-hoc sub-group analysis were progression of drusen number, total diameter, and total area on fundus photography, and their association with DHA supplementation, socio-demographic and genetic characteristics.

**Results:**

Drusen progression was analyzed in 167 subjects that did not develop CNV (87 that received DHA and 80 that received placebo). None of the drusen remodeling outcomes were significantly associated with DHA supplementation. Total drusen diameter reduction in the inner subfield was significantly associated with age (older patients: r = -0.17; p = 0.003). Women showed a tendency to decreased total drusen diameter in the inner subfield with *CFH* polymorphism (p = 0.03), where women with TT genotype tended to have a greater reduction in drusen diameter than other genotypes (CC and CT). Drusen area in the inner subfield was more reduced in older patients (r = -0.17) and in women (p = 0.01). Drusen number showed no significant trends.

**Conclusions:**

Dynamic drusen remodeling with net reduction in drusen load over three years was found in patients with exudative AMD in one eye and drusen in the other eye (study-eye). This reduction was correlated with increased age and female gender, and showed a tendency to be influenced by *CFH* genotype, but did not appear to be affected by DHA supplementation.

**Trial Registration:**

Controlled-Trials.com ISRCTN98246501

## Introduction

Age-related macular degeneration (AMD) is the leading cause of irreversible vision loss in patients over 50 [1]. Early stage AMD is asymptomatic and characterized by the presence of macular drusen and retinal pigment epithelium (RPE) changes. Drusen are characterized as hard or soft, as well as small (<63 μm), intermediate (>63 μm but <125 μm), or large (>125 μm) [2,3]. Different population-based studies and clinical trials have indicated that large, soft, confluent drusen are associated with a greater risk for developing advanced AMD [2,4,5]. However, as described in clinical and histopathologic studies, large soft macular drusen may even spontaneously regress [2,6,7,8,9,10].

Almost 80% of AMD patients with vision loss have exudative AMD [11], one of the two forms of advanced AMD (the other being the atrophic), which is characterized by the development of choroidal neovascularization (CNV). When the first eye is affected by exudative AMD, the probability of occurrence of CNV in the fellow eye is around 10–12% per year. There is consistent evidence that high intake of docosahexaenoic acid (DHA; 22:6, n-3), a long-chain omega-3 polyunsaturated fatty acid (PUFA) present in oily fish, is associated with a reduced risk of neovascular AMD [12–18]. In the Nutritional AMD treatment-2 (NAT-2) study [19,20], we hypothesized that targeting lipid metabolism in AMD may be a way of preventing the CNV development.

DHA is an essential fatty acid that, with dietary intake, is incorporated into circulating lipids and body cells along with EPA (Eicosapentaenoic Acid) [21]. It exerts numerous biological effects in vessels and tissues through signal transduction, gene regulation and plasma membrane remodeling [22]. In the retina, DHA increases mitochondrial activity as well as anti-oxidative, anti-inflammatory, anti-apoptotic, and anti-angiogenic effects [23]. It may preserve retinal neuron function and promote their survival as shown in other organs with slow or low cell-renewal (e.g. the aging brain or the post-ischemic myocardium) [24,25,26].

DHA is a major lipid constituent (>50%) of photoreceptor membranes, where it plays a crucial role in maintaining their structural and functional integrity. The continuous renewal of retinal membranes requires a constant supply of omega-3 fatty acids by RPE cells. Diets rich in DHA may improve retinal function and delay the development of advanced AMD [14,17,23]. On the other hand, an imbalance in retinal lipids leads to photoreceptor degradation and accumulation of lipid and lipoprotein debris in the RPE layer and sub-RPE space (drusen, for example).

All these properties of omega-3, together with the dynamic nature of drusen, including regression of some drusen, and formation of new drusen (that can occur simultaneously in the same macula) [27,28], raise the possibility that treatments such as oral DHA supplements capable of altering drusen morphology may influence disease progression in AMD.

The aim of this study was to evaluate the effects of oral DHA supplement on dynamic remodeling of drusen in the NAT-2 study: a 3-year prospective, single-center, double-blinded, randomized, placebo-controlled trial.

## Methods

### Clinical Trial declaration

The study was declared to the “International Standard Randomized Controlled Trial Number Register” (after enrolment of participants started, as at that time such declaration was not mandatory) and was allocated ISRCTN98246501 registration number. The authors confirm that all ongoing and related trials for this drug/intervention are registered.

### Study Participants

Participants of the NAT-2 study were enrolled prospectively from December 2003 until October 2005 at a single center, the Department of Ophthalmology at the *Hôpital intercommunal de Créteil* (Créteil, France). Eligible subjects were male and female with neovascular AMD in one eye and age-related maculopathy (defined as any drusen with or without pigmentary changes) in the fellow eye (study eye; not affected by CNV at entry). Inclusion criteria were as follows: 1) age ≥55 and <85 years; 2) signed written informed consent; 3) visual acuity ≥+0.4 LogMAR units in the study eye; and 4) subjects likely to attend follow-up visits during the study period. The main exclusion criteria were as follows: 1) CNV in both eyes or no CNV; 2) wide central atrophy encroaching on the fovea of the study eye; 3) progressive ocular diseases (severe glaucoma or other severe retinopathy); 4) corneal or lens opacities precluding retinal evaluation; 5) serious systemic disease (cancer, stroke, etc.) preventing long-term participation; 6) known allergy to the substances used in the study; 7) anticoagulant therapy or bleeding tendency; 8) current or recent treatment with prohibited medications or nutritional supplements (Maxepa^®^, docosahexaenoic acid, oral supplement containing omega-3 fatty acids; alpha-tocopherol acetate) or any concomitant nutritional supplement; 9) participation in a clinical trial within the last 30 days; 10) history of drug abuse or excessive use of medication; 11) subjects likely to be lost to follow-up or unlikely to comply with the study protocol; 12) monocular subjects for reasons other than AMD; and 13) subjects not covered by the French National Health system or wards of court.

We obtained written informed consent from all participants involved in the study. Subjects were free to withdraw from the study at any time. They could also withdraw or be withdrawn if they experienced serious adverse events, if it was thought that continuing to participate in the study would compromise their health, or if they failed to comply with study protocol. The study was reviewed and approved by the relevant IRB, (16 janurary 2003—*Comité de Protection des Personnes*, Paris-Ile de France 5, Paris, France). It was conducted in compliance with local regulations and approved by the national advisory commission on databases computing personal information (*Commission Nationale Informatique et Libertés*). It complied with ICH GCP guidelines, and the Declaration of Helsinki (1975, revised in 2000).

### Study design

**Fig 1:**
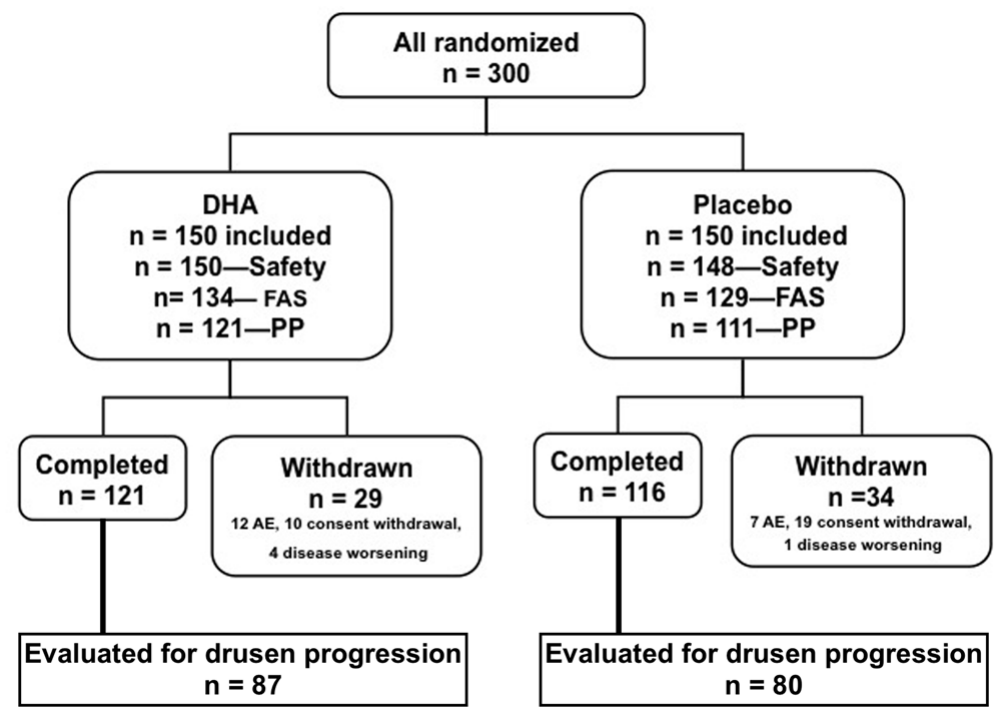
Diagram showing the study population. The safety population included all subjects who have received the study treatment. Full analysis set (FAS) included all subjects in the safety set who have had at least 1 post-baseline evaluation regarding occurrence of choroidal neovascularization. Per protocol (PP) population included all FAS subjects without major protocol deviation. Among those withdrawn there were 3 deaths in the docosahexaenoic acid (DHA) group and 6 deaths in the placebo group.

NAT-2 was a double-blinded, prospective, randomized, parallel, comparative trial following subjects with neovascular AMD in one eye who received oral DHA or placebo over 3 years (Fig 1).

### Intervention, randomization and blinding, and examination schedule

The study protocol has been previously described elsewhere [19]. In brief, eligible subjects were randomized in a 1:1 ratio to receive 3 capsules daily containing 280 mg DHA each (Reti-Nat^®^, provided by Bausch & Lomb), the effective DHA dose being 840 mg/day, or placebo (602 mg olive oil—since there is no fatty acid with a “neutral” biological effect, olive oil was selected as the placebo because it is part of a staple diet and has no deleterious effect on the retina) for 3 years on an out-patient basis. QL-Ranclin software (Qualilab, France) was used to generate the randomized list prior to enrollment. The subjects and the study personnel were both blinded to the treatment assignment.

Subjects were examined at baseline (Visit 1), 6 months (Visit 2), 1 year (Visit 3), 2 years (Visit 4), and 3 years (Visit 5). At baseline, clinical and ophthalmologic examinations of potentially eligible subjects were checked against inclusion/exclusion criteria. Data recorded included socio-demographic information, relevant ocular and medical history, and concomitant treatment. Biological samples were collected at baseline examination before any supplementation. They included serum lipids and lipoproteins and genetic polymorphisms validated as genetic markers of exudative AMD. Genomic DNA was extracted from 10 mL blood leukocytes as previously described in AMD patients [29] and using the Illustra^®^ kit according to the manufacturer’s protocol (GE Healthcare) in controls. Genotyping of *CFH* rs1061170 and *ARMS2/HTRA1* rs10490924 alleles were performed by quantitative polymerase chain reaction allelic discrimination using reagents and conditions from Custom Taqman Single-Nucleotide Polymorphism Genotyping Assays (Applera, Corp, France), using ABI 7900HT (Applied Biosystems). Quality control of genotyping by Sanger sequencing and bioinformatics analysis were performed as described [29].

The following ophthalmic examinations were performed at each visit: (1) best-corrected visual acuity, (2) intra-ocular pressure (IOP), (3) slit lamp examination, (4) fundus photography, and (5) fluorescein angiography (FA). FA was performed to screen for the presence of CNV or in subjects experiencing visual symptoms at any time during the study. Subjects with CNV were treated with laser, photodynamic therapy, or anti-VEGF intravitreal injections.

### Outcome Measures

The primary outcome of the current post-hoc sub-group analysis was drusen burden and progression, based on number, size, and area on fundus photography (dynamic remodeling of drusen) in subjects with neovascular AMD in one eye after receiving oral DHA or placebo over 3 years (i.e. different parameters were investigated in the same population of participants to the NAT-2 study [19]).

For the purpose of the study, the Safety Set included those subjects who received at least one unit of study medication, with at least one post-baseline value for progression to CNV. Safety was assessed by determining ocular and systemic tolerance to study treatment and included slit lamp examination, evaluation of lens opacity, and measurement of IOP. The Full Analysis Set (FAS) included all subjects in the Safety Set with at least one post-baseline assessment of CNV). The Per-Protocol Analysis Set (PPAS) included those patients in the FAS population without a major deviation from the protocol.

### Drusen Measurements and Dynamic Remodeling

**Fig 2:**
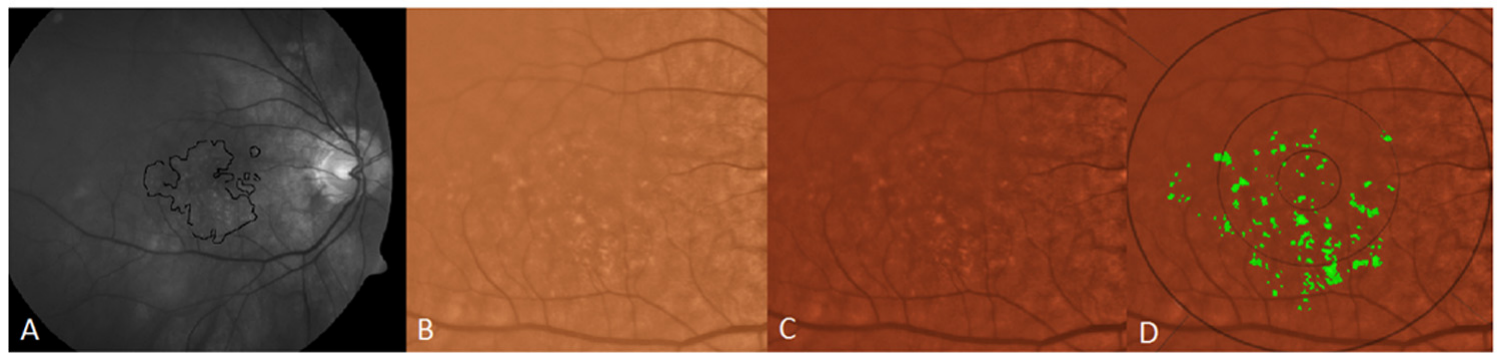
Illustration of drusen segmentation methods. A) Red-free fundus photo. Areas with drusen are circled in black (human supervision) to initialize the segmentation program. This allows the program to exclude from consideration peripapillary changes and other hypo-pigmented or hyper-reflective non-drusen features, resulting in a more rapid and accurate drusen identification. B) original color fundus photograph, cropped to 6 mm field C) contrast-enhanced color photograph D) contrast-enhanced color photograph with superimposed Wisconsin grading template (6 mm diameter circle with central, middle and outer subfields of diameters of 1, 3 and 6 mm). Drusen have been automatically segmented in detail (green) by the custom software within the areas containing drusen identified in A.

**Fig 3:**
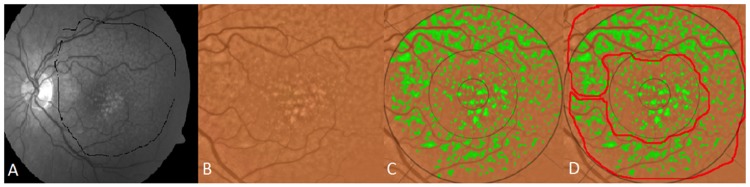
Segmentation of reticular pseudodrusen. A) original fundus photograph with large area containing both reticular macular disease (reticular pseudodrusen) and ordinary soft drusen outlined in black. The soft drusen are confined to the central macula. The reticular pseudodrusen extend to the arcades. B) original color fundus photo C) both reticular pseudodrusen and soft drusen are segmented (green) on the color photo within the area identified in A and within the superimposed Wisconsin grading template. D) The area of reticular macular disease is separately enclosed in red, and was excluded from drusen measurements.

**Fig 4:**
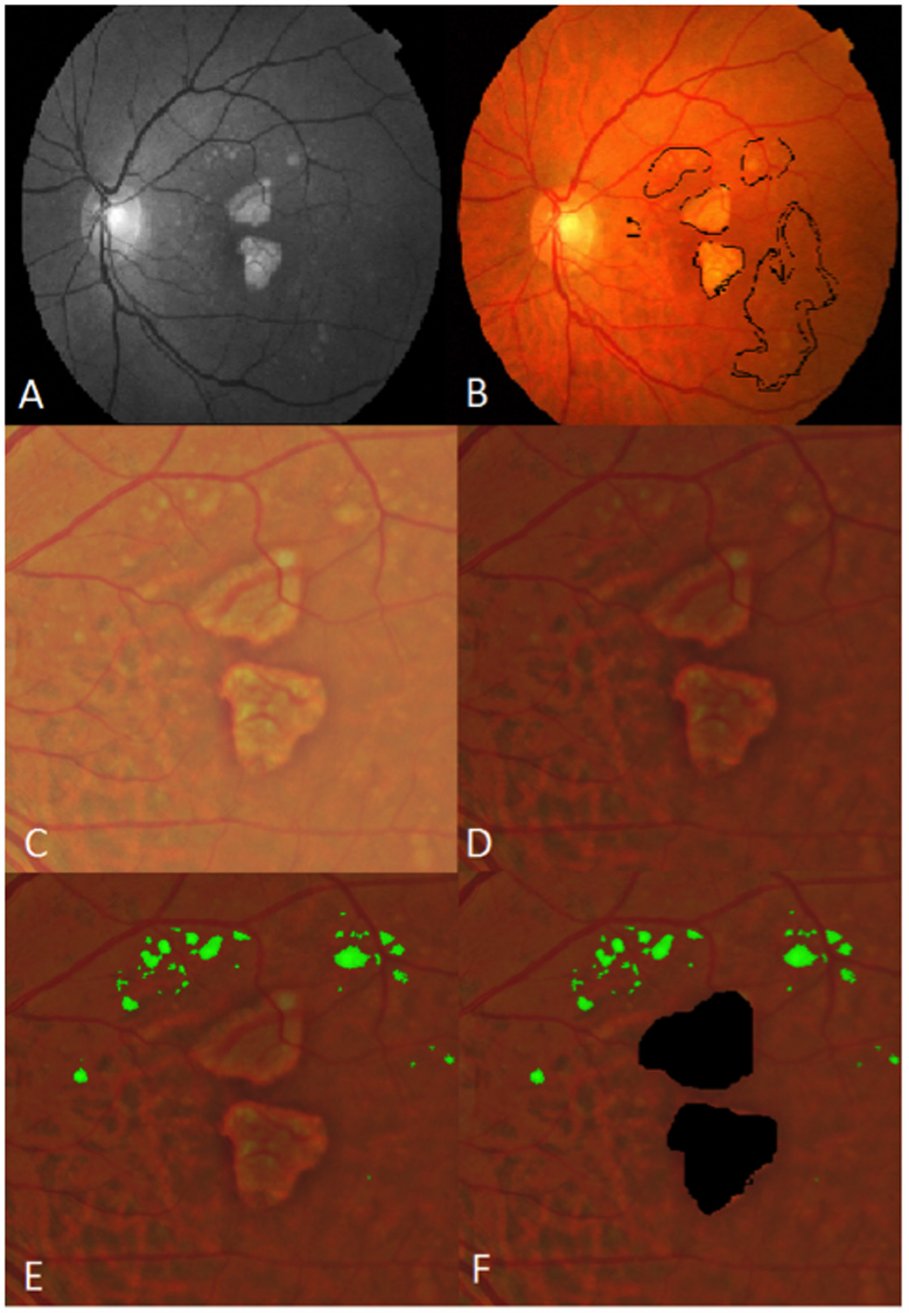
Segmentation of drusen and geographic atrophy (GA). A) Original red-free fundus photograph B) Original color fundus photograph with areas of drusen and GA outlined (black) C) Color fundus photograph cropped to 6 mm field. D) contrast enhanced color photo. E) segmentation of drusen (green) within the 6 mm field F) areas of GA masked (black).

**Fig 5:**
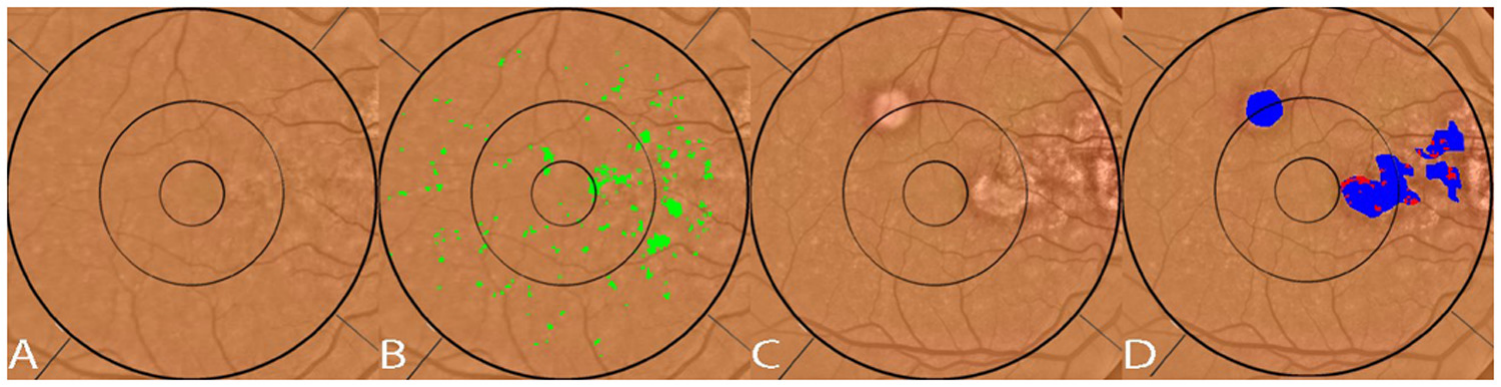
Drusen regression and its correlation with new geographic atrophy (GA). **A:** Color photo, right eye, Visit 1 (V1). **B:** V1 photo with soft drusen segmented in green, total pixel area 2,452. GA is not present. **C:** Color photo, right eye, Visit 5 (V5), showing new GA, **D:** V5 photo with GAs segmented in blue, total pixel area 4,161. Drusen from V1 that were absorbed in the GA are overlaid and segmented in red, total pixel area 381, or 9% of geographic atrophy total area. Compared to the original drusen area present in V1 of 2,452 pixels, the 381 drusen pixels converting to GA in V5 represent 16% of the original drusen area.

The region studied was a central 6000 mm diameter circle, with inner (<1000 μm diameter), middle (1000–3000 μm diameter) and outer (3000–6000 μm diameter) subfields defined by the Wisconsin grading template [30,31]. Drusen measurements from Visit 1 to Visit 5 were determined by fundus photography through the dilated pupil using a Topcon 501A CCD camera (Topcon, Tokyo, Japan) to record images centered on the macula. Uncompressed digital images in.tiff or.jpeg format were analyzed for quantification of drusen (Figs 2–5). After image enhancement, drusen were automatically detected, classified according to size and area, and quantified with sector localization as previously reported [30,31,32]. Before drusen segmentation, initial and final images were precisely registered as previously reported, with foveal co-localization and regions determined in an image stack by a single template in Adobe Photoshop (Photoshop 7.0, Adobe Systems Inc. San Jose, CA) for dynamic drusen remodeling assessment [28].

The segmentation and registration techniques, although quite detailed mathematically, may be briefly described. The main challenge with automated drusen segmentation is compensating for the variable reflectance and illumination of the image background. Our solution is a process known as background leveling, which involves correction of the macular image in multiple regions, exploiting the specific geometry of macular reflectance, and is also quite general, with applications beyond the analysis of ophthalmic images [US Patent # 7,248,736 B2, July, 2007] [33,34,35]. For fundus photographs, we demonstrated that our mathematical model, consisting of quadratic polynomials in several zones with cubic spline interpolation in blending regions between the zones, could model the global macular image background of a normal photograph with sufficient accuracy to allow its reconstruction and leveling [33,34,35]. Finally, background leveling for a drusen image was combined with the well-known, histogram-based Otsu method [36] for background selection of input to the model and final threshold selection to achieve a largely automated method of drusen segmentation (Fig 2) [37]. This algorithm also extends to segmentation of reticular pseudodrusen aka reticular macular disease (Fig 3), and to images with co-existent geographic atrophy (Fig 4). This model has been tested for accuracy and has also been implemented in autofluorescence images [35,38]. The model for automated drusen segmentation was developed within Matlab (Matlab 7.0; The Mathworks, Inc., Natick, MA) and the results are visualized in Adobe Photoshop, both of which are commercially available. We also applied it from a free-standing graphical user interface that does not require any other software.

For image registration and dynamic soft drusen remodeling measurements, each of the serial pairs of images was precisely registered by a completely automated technique based on a distinctive local feature descriptor (Intensity Invariant Feature Descriptor (IIFD)). These non-rigid transformations have been extensively validated on multimodal images, and are able to register an autofluorescence (AF), infrared (IR), color photographic or fluorescein angiographic (FA) image of a given eye to another such image of any of these types (AF to IR, color to FA, etc). The foveal locations then correspond, and a single circular template may be placed on the registered image stack in Photoshop for further analysis. In the case at hand, drusen segmentation on the initial and final color images was then performed as just described. Dynamic drusen remodeling measurements were made from the segmented images [28]. Thus, the dynamic change in drusen area was D1—D0, where D1 is the drusen area in the final image, and D0 is the drusen area in the baseline image. Drusen number and total diameter remodeling were measured analogously. Drusen remodeling variables of area, diameter and number were also calculated for each location (the inner, middle, and outer subfields, and the <6000 μm diameter field).

Geographic atrophy measurements were made from the same segmented images as analysis of drusen remodeling. Color fundus images from the final visit of patients with geographic atrophy served as a background on which an image stack in Photoshop was created. The images stacked were the drusen segmentation from the first visit, as determined by previously described methods, and highlighted atrophic areas, as determined by a single operator and reviewed by a second observer to limit intra-observer biases. Overlap placement of drusen from the first visit that were areas of atrophy by the final visit were highlighted in a third stacked layer in Photoshop. Measurements for pixel areas for each of these three layers were obtained and analyzed.

### Statistical Analyses

Dynamic remodeling of drusen was only assessed in the population of subjects where CNV was not detected using high quality fundus photography in the study eye during the study duration. Therefore, drusen progression was analyzed in those subjects that did not develop CNV in the PPAS population, which included only the subjects in the FAS population (who received at least one unit of study medication, with at least one post-baseline evaluation) without a major deviation from the protocol, and not in all subjects in the Safety Set.

Difference in age and drusen characteristics (number, diameter and area on different locations, inner, middle, outer and all (<6000 μm diameter)) at baseline was compared for the two treatment groups (DHA vs. placebo) using Student’s t Test. Drusen characteristics variables were previously log transformed (log(Y+c)). Difference for gender, smoking and *CFH* and *ARMS2* polymorphims between DHA and placebo group was assessed by Chi² test. (Table 1).

**Table 1:** Socio-demographic, genetics and drusen characteristics at baseline.

Baseline characteristics	DHA group N = 87	Placebo group N = 80	P*
Age, (years) mean ± SD	74.4 ± 6.7	72.8 ± 6.9	0.13
Gender, n (%)			0.09
Men	28 (32.2)	36 (45.0)	
Women	59 (67.8)	44 (55.0)	
Smoking, n (%)			0.43
Ever smoker	35 (40.2)	37 (46.2)	
Never smoker	52 (59.8)	43 (53.8)	
Genetic polymorphisms			
CFH, n (%)			0.21
CC	21 (24.4)	27 (33.8)	
CT	46 (53.5)	32 (40.0)	
TT	19 (22.1)	21 (26.2)	
ARMS2, n (%)			0.72
CC	24 (27.9)	27 (33.8)	
CT	41 (47.7)	35 (43.7)	
TT	21 (24.4)	18 (22.5)	
Drusen characteristics, median (5^th^-95^th^)			
Inner <1000 μm diameter			
Drusen number, n	6 (0–15)	6 (0–20)	0.23
Drusen diameter, μm	139 (0–342)	173 (0–338)	0.84
Drusen area, μm²	47325 (0–288,066)	74074 (0–281,207)	0.39
Middle 1000–3000 μm diameter			
Drusen number, n	29 (0–134)	39 (1–142)	0.37
Drusen diameter, μm	372 (118–799)	404 (30–823)	0.80
Drusen area, μm²	340,192 (34,294–1,572,016)	403,120 (2915–1,672,839)	0.55
Outer 3000–6000 μm diameter			
Drusen number, n	42 (0–259)	47 (1–360)	0.67
Drusen diameter, μm	419 (0.0–1226)	446 (25–1370)	0.73
Drusen area, μm²	434156 (0–3,705,761)	490912 (1543–4,628,429)	0.70
<6000 μm diameter			
Drusen number, n	87 (10–368)	94 (8–497)	0.58
Drusen diameter, μm	902 (299–2232)	1075 (198–2284)	0.91
Drusen area, μm²	895404 (96,364–5,383,744)	1261145 (52,812–5,673,182)	0.81

* p for Student’s t test for age and drusen characteristics, Chi² for gender, smoking, CFH and ARMS2 polymorphisms.

We compared drusen characteristics between baseline and 3 years according to treatment group (DHA vs. placebo) using paired T test. (Table 2).

**Table 2:** Comparison of drusen characteristics between baseline and 3 years according to treatment group.

	DHA group N = 87		p*	Placebo group N = 80		p*
Drusen characteristics, median (5^th^-95^th^)	Baseline	3 years		Baseline	3 years	
Inner <1000 μm diameter						
Drusen number, n	6 (0–15)	5 (0–17)	*0*.*21*	6 (0–20)	8 (0–18)	*0*.*94*
Drusen diameter, μm	139 (0–342)	158 (0–392)	*0*.*20*	173 (0–338)	192 (0–346)	*0*.*62*
Drusen area, μm²	47325 (0–288066)	61728 (0–378258)	*0*.*17*	74074 (0–281207)	91049 (0–294925)	*0*.*61*
Middle 1000–3000 μm diameter						
Drusen number, n	29 (0–134)	36 (0–116)	*0*.*58*	39 (1.0–142)	52 (1–126)	*0*.*23*
Drusen diameter, μm	372 (118–799)	448 (0–931)	*0*.*14*	404 (30–823)	481 (14–841)	*0*.*82*
Drusen area, μm²	340192 (34294–1572016)	494856 (0–2134774)	*0*.*12*	403120 (2915–1672839)	570645 (1019–1743141)	*0*.*85*
Outer 3000–6000 μm diameter						
Drusen number, n	42 (0–259)	42 (0–267)	*0*.*45*	47 (1–360)	59 (0–318)	*0*.*69*
Drusen diameter, μm	420 (0–1226)	383 (0–1303)	*0*.*52*	446 (25–1370)	391 (0–1452)	*0*.*38*
Drusen area, μm²	434156 (0–3705761)	361111 (0–4185871)	*0*.*51*	490912 (1543–4628429)	376200 (0–5203532)	*0*.*36*
<6000 μm diameter						
Drusen number, n	87 (10–368)	87 (0–396)	*0*.*61*	94 (8–497)	114 (9–450)	*0*.*26*
Drusen diameter, μm	902 (299–2232)	1155 (0–2332)	*0*.*28*	1075 (198–2284)	1156 (143–2402)	*0*.*67*
Drusen area, μm²	895404 (96364–5383744)	1411523 (0–5385459)	*0*.*30*	1261145 (52812–5673182)	1421982 (28121–63617966)	*0*.*69*

* p for paired T test. Drusen characteristics were log transformed (log(Y+c)).

Associations of DHA supplementation with drusen remodeling were estimated using linear regression adjusted for age and gender. 3-year drusen remodeling data were used as the dependent variables and the treatment group (DHA or placebo), age and gender as the independent variables.

Because we studied 3 drusen characteristics in 4 different locations, we used the Bonferroni’s correction for multiple tests; p<0.005 was considered as significant. (Table 3).

**Table 3:** Associations of DHA supplementation with drusen remodeling.

	Difference between the first and the last visit			
	DHA group N = 87	Placebo group N = 80	Difference between groups (95% CI)	P*
	mean ± SD	mean ± SD		
Inner <1000 μm diameter				
Drusen number, n	-0.03 ± 6.40	0.73 ± 10.00	0.57 (-2.02;3.17)	0.66
Drusen diameter, μm	21.00 ± 121.90	7.39 ± 115.50	-25.43 (-61.32;70.46)	0.16
Drusen area, μm²	36 001 ± 126 720	14 239 ± 109 534	-34 735 (-70 316;845)	0.06
Middle 1000–3000 μm diameter				
Drusen number, n	0.23 ± 40.40	3.64 ± 34.90	3.00 (-8.84;14.84)	0.62
Drusen diameter, μm	32.40 ± 245.40	35.64 ± 231.60	-6.94 (-81.11;67.23)	0.85
Drusen area, μm²	123 839 ± 657 112	88 074 ± 569 970	-60 352 (-251 285;130 581)	0.53
Outer 3000–6000 μm diameter				
Drusen number, n	-2.77 ± 80.67	-4.24 ± 63.69	1.04 (-21.63;23.71)	0.93
Drusen diameter, μm	18.15 ± 310.30	26.67 ± 259.80	6.33 (-83.57;96.23)	0.14
Drusen area, μm²	120 760 ± 1 253 045	167 941 ± 991 149	31 998 (-323 188;387 185)	0.18
<6000 μm diameter				
Drusen number, n	-2.57 ± 115.30	0.13 ± 90.71	4.61 (-27.88;37.10)	0.28
Drusen diameter, μm	71.51 ± 579.60	69.70 ± 484.10	-26.05 (-192.62;140.53)	0.76
Drusen area, μm²	280 600 ± 1 773 172	270 254 ± 1 356 409	-63 089 (-55 417;431 240)	0.80

* p for linear regression adjusted for age and gender

Associations of 3-year drusen number, diameter and area remodeling in each location with socio-demographic and genetics characteristics were estimated using Pearson correlation for age, Student’s t Test for gender and smoking and ANOVA for *CFH* and *ARMS2* polymorphisms. (Table 4).

**Table 4:** Associations of 3-year drusen number, diameter and area remodeling with socio-demographic and genetics characteristics at baseline.

3-years Drusen Number, remodeling	Inner <1000 μm diameter	Middle 1000–3000 μm diameter	Outer 3000–6000 μm diameter	<6000 μm diameter
Age	r = -0.60	r = 0.1	r = 0.13	r = 0.09
Pearson’s correlation	p = 0.42	p = 0.86	p = 0.09	p = 0.23
Gender				
Men n = 64	0.64 ± 7.47	4.73 ± 39.91	-3.38 ± 69.70	2.00 ± 102.9
Women n = 103	0.14 ± 8.78	0.08 ±36.49	-3.53 ± 75.03	-3.32 ± 105.0
p T test	0.70	0.44	0.99	0.75
Smoking				
Ever smoker n = 72	0.37 ± 8.83	-0.65 ± 39.72	-7.33 ± 65.35	-7.71 ± 94.83
Never smoker n = 95	0.28 ± 7.56	3.76 ± 36.35	-0.55 ± 78.22	3.59 ± 110.6
P T test	0.94	0.46	0.55	0.49
Genetic polymorphisms				
CFH				
CC n = 48	2.08 ± 10.94	7.58 ± 31.71	-0.69 ± 36.57	8.98 ± 64.41
CT n = 78	-0.65 ± 7.50	0.14 ± 38.06	0.54 ± 87.04	0.03 ± 120.04
TT n = 40	0.30 ± 5.47	-0.18 ± 43.20	-11.20 ± 73.56	-11.08 ± 106.07
P anova	0.20	0.51	0.69	0.66
ARMS2				
CC n = 51	1.73 ± 1.73	6.18 ± 6.18	5.53 ± 5.53	13.43 ± 13.43
CT n = 76	-0.54 ± 7.41	0.80 ± 34.97	-6.17 ± 63.06	-5.91 ± 91.82
TT n = 39	0.36 ± 7.34	-0.21 ± 40.86	-6.46 ± 88.93	-6.31 ± 122.33
P anova	0.32	0.66	0.63	0.53
3-years Drusen diameter, remodeling	Inner <1000 μm diameter	Middle 1000–3000 μm diameter	Outer 3000–6000 μm diameter	<6000 μm diameter
Age	r = -0.17	r = -0.12	r = 0.004	r = 0.09
Pearson’s correlation	p = 0.003	p = 0.11	p = 0.96	p = 0.24
Gender				
Men n = 64	39.50 ±106.3	44.51 ± 229.9	34.21 ± 271.5	118.2 ±474.7
Women n = 103	-1.07 ± 123.8	27.36 ± 244.1	14.79 ± 296.3	41.08 ±568.5
p T test	0.03	0.65	0.67	0.35
Smoking				
Ever smoker n = 72	14.84 ± 114.4	12.88 ± 234.4	23.85 ± 255.8	51.57 ± 468.4
Never smoker n = 95	14.21 ± 122.5	49.88 ± 241.1	21.01 ± 308.9	85.10 ± 581.5
P T test	0.97	0.32	0.95	0.68
Genetic polymorphisms				
CFH				
CC n = 48	0.36 ± 122.45	25.36 ± 209.11	-19.33 ± 253.95	6.39 ± 493.40
CT n = 78	2.14 ± 115.73	16.59 ± 242.68	33.79 ± 293.19	52.51 ± 534.01
TT n = 40	57.69 ± 113.29	79.36 ± 264.00	66.49 ± 292.53	203.53 ± 564.03
P anova	0.03	0.39	0.35	0.20
ARMS2				
CC n = 51	14.77	33.78	56.65	105.20
CT n = 76	7.29	28.02	7.57	42.87
TT n = 39	30.37	47.02	23.15	100.54
P anova	0.62	0.92	0.63	0.77
3-years Drusen area, remodeling	Inner <1000 μm diameter	Middle 1000–3000 μm diameter	Outer 3000–6000 μm diameter	<6000 μm diameter
Age	r = -0.17	r = -0.15	r = -0.04	r = -0.10
Pearson’s correlation	p = 0.03	p = 0.06	p = 0.62	p = 0.21
Gender				
Men n = 64	54774 ± 120207	102543 ± 614354	164699 ± 1204769	322016 ± 1585241
Women n = 103	7434 ± 115034	109293 ± 618919	130103 ± 1090247	246830 ± 1587965
p T test	0.01	0.95	0.85	0.77
Smoking				
Ever smoker n = 72	30307 ± 120989	55117.7 ± 646690	203588 ± 1214619	289013 ± 1639605
Never smoker n = 95	21990 ± 117889	145805 ± 590911	97716 ± 1069527	265511 ± 156636
P T test	0.66	0.35	0.55	0.93
Genetic polymorphisms				
CFH				
CC n = 48	12787 ± 115886	113726 ± 440820	26393 ± 898461	152905 ± 1223983
CT n = 78	14017 ± 117012	20559 ± 658430	91726 ± 1200843	126301 ± 1683038
TT n = 40	65895 ± 120930	270413 ± 691643	451980 ± 1152212	788289 ± 1644371
P anova	0.05	0.11	0.16	0.07
ARMS2				
CC n = 51	23024	90959	266192	380174
CT n = 76	26505	91970	101243	219718
TT n = 39	29596	160265	134114	323976
P anova	0.97	0.83	0.71	0.85

Statistical analyses were performed using SAS^®^ 9.2 (SAS Institute Inc., Cary, NC, USA).

## Results

### Data Sets Analyzed

Among the 300 randomized subjects, a total of 298 subjects were included in the Safety Set (150 subjects in the DHA group and 148 in the placebo group). The FAS (all subjects in the safety set with at least one post-baseline assessment of CNV) consisted of 134 subjects in the DHA group and 129 in the placebo group. After exclusion of subjects with major deviations (mainly for premature withdrawal), the PPAS, those in the FAS population without a major deviation from the protocol that could jeopardize the primary outcome, included 121 subjects in the DHA group and 111 in the placebo group (Fig 1). In the PPAS population, drusen progression was analyzed in those subjects that did not develop CNV, counting for overall 167 patients, 87 that received DHA and 80 that received placebo.

### Socio-demographic and Other Baseline Characteristics

Table 1 shows baseline characteristics in the two treatment groups. DHA and placebo groups were not statistically different in age (p = 0.13), gender (p = 0.09), smoking history (p = 0.95) or genetic polymorphisms for *CFH* and *ARMS2* (p = 0.22 and p = 0.53, respectively). At baseline, drusen characteristics (i.e. drusen number, drusen diameter, drusen area) in each location (inner, middle, outer subfields, and the <6000 μm diameter field) were not statistically different between DHA and placebo group (p = 0.33 to p = 0.95).

### Occurrence and progression of drusen

Table 2 shows comparison of drusen characteristics between baselin and 3 years. None of the drusen characteristics, in any location, differed significantly after 3-years DHA-supplementation.

Table 3 shows associations of DHA supplementation with drusen number, diameter and area remodeling in the inner, middle, and outer subfields and the <6000 μm diameter field. None of the drusen remodeling features, in any location, were significantly associated with DHA supplementation.

Table 4 shows associations of 3-year drusen number, diameter and area remodeling with socio-demographic and genetic characteristics.

Drusen number remodeling was not significantly associated with age, gender, smoking, *CFH* or *ARMS2* polymorphisms in any location. Drusen diameter remodeling in the inner subfield was significantly associated only with age: total drusen diameter in the inner subfield was more reduced in older patients (r = -0.17; p = 0.003). Women tended to show a greater reduction in total drusen diameter in the inner subfield with the *CFH* polymorphism (TT) genotype than with the heterozygous or homozygous risk alleles (CT\CC) (p = 0.03), Concerning drusen area remodeling, we found that drusen area in the inner subfield was also reduced in older patients (r = -0.17) and in women (p = 0.01), but these associations did not reach statistical significance. No association was found between any drusen remodeling variable in the middle or outer subfields or the <6000 μm diameter field and any socio-demographic and genetic characteristics (Table 4).

We also analyzed drusen remodeling according to EPA and DHA measured in serum and in red-blood cell membranes. We found no significant results (data not shown).

Additionally, we analyzed drusen remodeling in concordance with development of geographic atrophy. Of the 167 patients that were analyzed for drusen progression, 16 patients had observer determined geographic atrophy develop by their final visit. From this subset, we obtained measurements of both the area of drusen present initially and geographic atrophy present finally along with their areas of coincidence (Fig 5). The average initial total pixel area of drusen in these subjects was 7,062.47. The average total pixel area of geographic atrophy at the last visit was 10,585.07. However, the average proportion of this geographic atrophy resulting from former areas of drusen was only 13%. Conversely, only 15% of the total drusen area present in the first visit transformed into areas of atrophy.

## Discussion

Drusen in AMD are dynamic with regression of some drusen and formation of new drusen (that can occur simultaneously in the same macula) [27,28]. DHA is a major lipid constituent (>50%) of photoreceptor membranes, where it plays a crucial role in maintaining their structural and functional integrity. An imbalance in retinal lipids may lead to photoreceptor degradation and accumulation of lipids and lipoprotein debris in the RPE layer and sub-RPE space (drusen, for example). For these reasons, in this post-hoc sub-group analysis we investigated whether DHA supplements might be capable of altering drusen morphology and influence AMD progression in a 3-year prospective, single-center, double-blinded, randomized, placebo-controlled trial. We selected a homogeneous group of 300 patients affected with exudative AMD in one eye and drusen in the study eye. Both treatment groups were comparable for socio-demographic characteristics and ocular characteristics of the study eye. Other clinical features (age, sex ratio, smoking status, family history) were characteristic of an exudative AMD population. DHA was given at a daily dose of 840 mg in the form of fish-oil containing a DHA:EPA ratio of 3:1 (3 capsules, equivalent to eating approximately 100 g of oily fish/day) for 3 years. This dose (1100 mg EPA+DHA) is about twice the recommended nutritional daily intake of EPA+DHA (500 mg) in France [39], and corresponds to the total daily dose—although with a higher DHA/EPA ratio—proposed in large randomized controlled trials using omega-3 fatty acids in the prevention of cardiovascular disease [31].

Overall, in our study, there was no significant difference in the progression of drusen number over three years between groups; of note a slight decrease in the drusen number in both groups in the outer diameter, and in the DHA group in the inner, outer and <6000 μm diameter (Table 2). Also, the progression of the total diameter and area of drusen did not differ between groups.

The NAT-2 study primary outcome was incidence of CNV in the study eye between groups. Oral DHA did not significantly modify the incidence of CNV over 3 years compared to placebo [19]. Similarly, in the current post-hoc sub-group analysis, DHA did not significantly modify drusen remodeling over 3 years compared to placebo.

There have been several observational studies that suggest that nutritional interventions may reduce the incidence of AMD [17,18,40,41,42,43]. Previous open-label studies also support the hypothesis that oral DHA supplementation may play a beneficial role in prevention of AMD [44,45]. On the other hand, the recently published large, phase 3, Age-Related Eye Disease Study 2 (AREDS2) [46], a follow-up study from AREDS1, evaluated the effect of carotenoids lutein and zeaxanthin as well as omega-3 fatty acids DHA and EPA on rate of progression to advanced AMD and/or moderate vision loss in people at moderate to high risk for progression. In contrast to evidence from most of the related literature, AREDS2 findings suggest that omega-3 does not prevent progression of AMD. To our knowledge, the NAT-2 study was the first randomized double-blind study exploring the potential role of a long-term oral PUFA supplement enriched in DHA over placebo in preventing CNV development and influencing dynamic drusen remodeling in a homogenous group of patients with a typical and severe form of AMD. Overall we found that the progression of total number, diameter and area of drusen did not differ between DHA and placebo groups.

It is believed that drusen disappearance is generally beneficial for the disease, but there are significant exceptions, as in the case of drusen regression followed by GA. Recent studies using color fundus photography have demonstrated spontaneous drusen regression; however, in conjunction with regression, new drusen in other locations may appear and grow in size [8,9,47]. To date, it is not completely known how the processes of drusen creation and absorption are related temporally and spatially, or to what extent they may be mutually exclusive or can occur simultaneously [28]. In our subset of patients who developed geographic atrophy, only a minority of the amount of drusen found in their first visit, 15%, was involved in GA development. Additionally, in the total amount of geographic atrophy only 13% was attributable to drusen regression. Thus, drusen regression in our study population, appeared to be largely a benign development. Further, drusen remodeling can be studied as an individual phenomenon or a group phenomenon. The purpose of averaging together (as done herein) the findings in a large group is to seek significant results that will have broad applicability. However, as evidenced by the large standard deviations in the measurements, there are clearly subpopulations with widely differing drusen remodeling. To address this, multivariate modeling was used to search for possible determinants of this variation such as gender, AMD risk allele status, etc. Indeed, many trends were found: *all* indices of drusen remodeling were *more* decreased in the central 1000 μm circle for women than men, and with increasing age. However, only decreasing drusen total diameter in this region with age was significant. Variability in a single individual is clearly not predictable. Furthermore, the relationship between drusen indices and disease progression can be quite complex. Thus, with evident disease progression as measured by growing drusen area, drusen coalescing could actually cause the measured drusen number to decrease. Therefore, care is indicated in the interpretation of all such data.

A potential criticism of this study could be the lack of corroborative imaging with spectral domain optical coherence tomography (SD-OCT), which has been used for drusen volume endpoints. However, there is little evidence that drusen volume is superior to drusen area as a metric for disease. In any case, they largely track together. The methodologies are closely correlated as metrics and in drusen detection capability are essentially equivalent: Interestingly, there are also specific drusen morphologies that are better detected with one method or the other. Nevertheless, precise data on drusen number, area, and diameter with a validated method as used herein have sufficiently addressed these questions.

DHA has direct anti-angiogenic properties in the retina [48]. Our previous findings support a direct anti-angiogenic role of DHA [19], whereas the current findings do not support a role for DHA in slowing drusen occurrence and later AMD.

Our study has several limitations. Of the 300 enrolled subjects, only 167 patients (87 that received DHA and 80 that received placebo) in the PPAS population that did not develop CNV were analyzed for drusen progression, mainly because of patients’ decision or other health-related constraints leading to interruption of their participation to the study before the first follow-up visit. This may have reduced the statistical power for detecting drusen progression and differences between the two groups. However, since percentage of subjects, and reasons for interrupting were similar in both groups, interruption is however unlikely to have introduced a bias in the estimation of the difference between the two groups. In addition, three years of follow up may have been too short to study drusen progression. Insufficient statistical power may explain the lack of association between DHA supplementation and drusen remodeling in this subsample. This is reflected by the wide of confidence intervals. For example, regarding drusen number in the <6000 μm diameter field (Table 3), we cannot exclude that the true difference in drusen number may be as high as 37 (that is about 39% difference) between the placebo group compared to the DHA group. In conclusion, significant drusen dynamic changes in patients with exudative AMD in one eye and macular drusen in the other eye (study eye) over three years were only apparent in the inner 1mm subfield, and these all showed reductions in drusen load. Thus, a total drusen diameter reduction in the inner subfield was significantly associated with age. Women tended to show a greater reduction in total drusen diameter in the inner subfield with the *CFH* polymorphism TT genotype than with the heterozygous or homozygous risk genotypes. Drusen area in the inner subfield tended to show greater reductions in older patients and in women. DHA supplementation at a daily dose of 840 mg over 3 years in these patients had no effect over placebo on dynamic changes in overall drusen number, total diameter, and area. In summary, spontaneous dynamic drusen remodeling with net reduction in drusen load was found in the central macula. This reduction was correlated with increased age and female gender, and showed a tendency to be influenced by *CFH* genotype, but did not appear to be affected by DHA supplementation. Whether drusen load can be safely reduced by other interventions remains an open question.

## Supporting Information

S1 CONSORT Checklist CONSORT Checklist. (DOC)

S1 Protocol Trial protocol. (DOC)

## References

[1] Resnikoff S, Pascolini D, Etya'ale D, Kocur I, Pararajasegaram R, Pokharel GP, et al Global data on visual impairment in the year 2002. Bull World Health Organ. 2004; 82(11):844–851. 15640920 PMC2623053

[2] Ferris FL, Davis MD, Clemons TE, Lee LY, Chew EY, Lindblad AS, et al A simplified severity scale for age-related macular degeneration: AREDS Report No. 18. Arch Ophthalmol. 2005;123(11):1570–1574. 16286620 10.1001/archopht.123.11.1570 PMC1473206

[3] Seddon JM, Sharma S, Adelman RA. Evaluation of the clinical age-related maculopathy staging system. Ophthalmology. 2006;113(2):260–266. 16458093 10.1016/j.ophtha.2005.11.001

[4] Pauleikhoff D, Barondes MJ, Minassian D, Chisholm IH, Bird AC. Drusen as risk factors in age-related macular disease. Am J Ophthalmol.1990;109(1): 38–43. 1688685 10.1016/s0002-9394(14)75576-x

[5] Klein R, Klein BE, Knudtson MD, Meuer SM, Swift M, Gangnon RE. Fifteen-year cumulative incidence of age-related macular degeneration: the Beaver Dam Eye Study. Ophthalmology. 2007;114(2): 253–262. 17270675 10.1016/j.ophtha.2006.10.040

[6] Gass JD. Drusen and disciform macular detachment and degeneration. Arch Ophthalmol. 1973;90: 206–217. 4738143 10.1001/archopht.1973.01000050208006

[7] Sarks JP, Sarks SH, Killingsworth MC. Evolution of geographic atrophy of the retinal pigment epithelium. Eye (Lond). 1988;2 (Pt 5):552–77. 2476333 10.1038/eye.1988.106

[8] Bressler NM, Munoz B, Maguire MG, Vitale SE, Schein OD, Taylor HR. Five-year incidence and disappearance of drusen and retinal pigment epithelial abnormalities. Waterman study. Arch Ophthalmol. 1995; 113(3): 301–308. 7534060 10.1001/archopht.1995.01100030055022

[9] Sparrow JM, Dickinson AJ, Duke AM, Thompson JR, Gibson JM, Rosenthal AR. Seven year follow-up of age-related maculopathy in an elderly British population. Eye (Lond). 1997;11 (Pt 3):315–24. 9373468 10.1038/eye.1997.67

[10] Complications of Age-Related Macular Degeneration Prevention Trial Research Group. Laser treatment in patients with bilateral large drusen: the complications of age-related macular degeneration prevention trial. Ophthalmology. 2006;113(11): 1974–1986. 17074563 10.1016/j.ophtha.2006.08.015

[11] Hyman L, Neborsky R. Risk factors for age-related macular degeneration: an update. Curr Opin Ophthalmol. 2002;13(3): 171–175. 12011686 10.1097/00055735-200206000-00007

[12] Chong EW, Kreis AJ, Wong TY, Simpson JA, Guymer RH. Dietary omega-3 fatty acid and fish intake in the primary prevention of age-related macular degeneration: a systematic review and meta-analysis. Arch Ophthalmol. 2008;126(6): 826–833. 10.1001/archopht.126.6.826 18541848

[13] Augood C, Chakravarthy U, Young I, Vioque J, de Jong PT, Bentham G, et al Oily fish consumption, dietary docosahexaenoic acid and eicosapentaenoic acid intakes, and associations with neovascular age-related macular degeneration. Am J Clin Nutr. 2008;88(2): 398–406. 18689376 10.1093/ajcn/88.2.398

[14] Smith W, Mitchell P, Leeder SR. Dietary fat and fish intake and age-related maculopathy. Arch Ophthalmol. 2000;118(3): 401–404. 10721964 10.1001/archopht.118.3.401

[15] Cho E, Hung S, Willett WC, Spiegelman D, Rimm EB, Seddon JM, et al Prospective study of dietary fat and the risk of age-related macular degeneration. Am J Clin Nutr. 2001;73(2): 209–218. 11157315 10.1093/ajcn/73.2.209

[16] Merle B, Delyfer MN, Korobelnik JF, Rougier MB, Colin J, Malet F, et al Dietary omega-3 fatty acids and the risk for age-related maculopathy: the Alienor Study. Invest Ophthalmol Vis Sci. 2001;52(8): 6004–6011. 10.1167/iovs.11-7254 21705687

[17] Seddon JM, Rosner B, Sperduto RD, Yannuzzi L, Haller JA, Blair NP, et al Dietary fat and risk for advanced age-related macular degeneration. Arch Ophthalmol. 2001;119(8): 1191–1199. 11483088 10.1001/archopht.119.8.1191

[18] Merle BM, Delyfer MN, Korobelnik JF, Rougier MB, Malet F, Féart C, et al High concentrations of plasma n3 fatty acids are associated with decreased risk for late age-related macular degeneration. J Nutr. 2013;143(4): 505–511. 10.3945/jn.112.171033 23406618

[19] Souied EH, Delcourt C, Querques G, Bassols A, Merle B, Zourdani A, et al Oral Docosahexaenoic Acid in the Prevention of Exudative Age-Related Macular Degeneration: The Nutritional AMD Treatment 2 Study. Ophthalmology. 2013;120(8): 1619–1631. 10.1016/j.ophtha.2013.01.005 23395546

[20] Merle BM, Benlian P, Puche N, Bassols A, Delcourt C, Souied EH, et al Circulating omega-3 Fatty acids and neovascular age-related macular degeneration. Invest Ophthalmol Vis Sci. 2014;55(3): 2010–2019. 10.1167/iovs.14-13916 24557349

[21] Arterburn LM, Hall EB, Oken H. Distribution, interconversion, and dose response of n-3 fatty acids in humans. Am J Clin Nutr. 2006;83(6Suppl): 1467S–1476S. 16841856 10.1093/ajcn/83.6.1467S

[22] Simopoulos AP. The importance of the omega-6/omega-3 fatty acid ratio in cardiovascular disease and other chronic diseases. Exp Biol Med (Maywood). 2008;233(6): 674–688. 18408140 10.3181/0711-MR-311

[23] SanGiovanni JP, Chew EY. The role of omega-3 long-chain polyunsaturated fatty acids in health and disease of the retina. Prog Retin Eye Res. 2005;24(1): 87–138. 15555528 10.1016/j.preteyeres.2004.06.002

[24] Bazan NG. Cellular and molecular events mediated by docosahexaenoic acid-derived neuroprotectin D1 signaling in photoreceptor cell survival and brain protection. Prostaglandins Leukot Essent Fatty Acids. 2009;81(2–3): 205–211. 10.1016/j.plefa.2009.05.024 19520558 PMC2756692

[25] Lukiw WJ, Bazan NG. Docosahexaenoic acid and the aging brain. J Nutr. 2008;138(12): 2510–2514. 10.3945/jn.108.096016 19022980 PMC2666388

[26] Pepe S. Effect of dietary polyunsaturated fatty acids on age-related changes in cardiac mitochondrial membranes. Exp Gerontol. 2005;40(5): 369–376. 15919588 10.1016/j.exger.2005.03.005

[27] Sallo FB, Rechtman E, Peto T, Stanescu-Segall D, Vogt G, Bird AC, et al Functional aspects of drusen regression in age-related macular degeneration. Br J Ophthalmol. 2009;93(10): 1345–1350. 10.1136/bjo.2008.150334 19535356

[28] Smith RT, Sohrab MA, Pumariega N, Chen Y, Chen J, Lee N, et al Dynamic soft drusen remodelling in age-related macular degeneration. Br J Ophthalmol. 2010;94(12): 1618–1623. 10.1136/bjo.2009.166843 20530179 PMC2948612

[29] Leveziel N, Souied EH, Richard F, Barbu V, Zourdani A, Morineau G, et al PLEKHA1-LOC387715-HTRA1 polymorphisms and exudative age-related macular degeneration in the French population. Mol Vis. 2007;13: 2153–2159. 18079691

[30] Klein R, Davis MD, Magli YL, Segal P, Klein BE, Hubbard L. The Wisconsin age-related maculopathy grading system. Ophthalmology. 1991;98(7): 1128–1134. 1843453 10.1016/s0161-6420(91)32186-9

[31] Marik PE, Varon J. Omega-3 dietary supplements and the risk of cardiovascular events: a systematic review. Clin Cardiol. 2009;32(7): 365–372. 10.1002/clc.20604 19609891 PMC6653319

[32] NIH Age-Related Macular Degeneration Fact Sheet. Available: http://report.nih.gov/nihfactsheets/Pdfs/AgeRelatedMacularDegeneration(NEI).pdf

[33] Smith RT, Nagasaki T, Sparrow JR, Barbazetto I, Klaver CC, Chan JK. A method of drusen measurement based on the geometry of fundus reflectance. Biomed Eng Online. 2003;18;2:10. 10.1186/1475-925X-2-10 PMC155640 12740042

[34] Smith RT, Nagasaki T, Sparrow JR, Barbazetto I, Koniarek JP, Bickmann LJ. Photographic patterns in macular images: representation by a mathematical model. J Biomed Opt. 2004;9(1): 162–172. 14715069 10.1117/1.1630604

[35] Smith RT, Koniarek JP, Chan J, Nagasaki T, Sparrow JR, Langton K. Autofluorescence characteristics of normal foveas and reconstruction of foveal autofluorescence from limited data subsets. Invest Ophthalmol Vis Sci. 2005;46(8): 2940–2946. 16043869 10.1167/iovs.04-0778 PMC2754769

[36] Otsu N. A threshold selection method from gray-level histograms: IEEE Transactions on Systems, Man, and Cybernetics. 1979.

[37] Smith RT, Chan JK, Nagasaki T, Ahmad UF, Barbazetto I, Sparrow J, et al Automated detection of macular drusen using geometric background leveling and threshold selection. Arch Ophthalmol. 2005;123(2): 200–206. 15710816 10.1001/archopht.123.2.200 PMC2884376

[38] Hwang JC, Chan JW, Chang S, Smith RT. Predictive value of fundus autofluorescence for development of geographic atrophy in age-related macular degeneration. Invest Ophthalmol Vis Sci. 2006;47(6): 2655–2661. 16723483 10.1167/iovs.05-1027 PMC2754747

[39] AFSSA. Avis de l'Agence Française de Sécurité Sanitaire des Aliments relatif à l'actualisation des apports nutritionnels conseillés pour les acides gras. Maisons-Alfort: AFSSA 2010.

[40] Seddon JM, Cote J, Rosner B. Progression of age-related macular degeneration: association with dietary fat, transunsaturated fat, nuts, and fish intake. Arch Ophthalmol. 2003;121(3): 1728–1737. 14662593 10.1001/archopht.121.12.1728 PMC8443211

[41] Christen WG, Schaumberg DA, Glynn RJ, Buring JE. Dietary omega-3 fatty acid and fish intake and incident age-related macular degeneration in women. Arch Ophthalmol. 2011;129(7): 921–929. 10.1001/archophthalmol.2011.34 21402976 PMC3134638

[42] Sangiovanni JP, Agron E, Meleth AD, Reed GF, Sperduto RD, Clemons TE, et al {omega}-3 Long-chain polyunsaturated fatty acid intake and 12-y incidence of neovascular age-related macular degeneration and central geographic atrophy: AREDS report 30, a prospective cohort study from the Age-Related Eye Disease Study. Am J Clin Nutr. 90(6): 1601–1607. 10.3945/ajcn.2009.27594 19812176 PMC2777471

[43] Chua B, Flood V, Rochtchina E, Wang JJ, Smith W, Mitchell P. Dietary fatty acids and the 5-year incidence of age-related maculopathy. Arch Ophthalmol. 2006;124(7): 981–986. 16832021 10.1001/archopht.124.7.981

[44] Cangemi FE. TOZAL Study: an open case control study of an oral antioxidant and omega-3 supplement for dry AMD. BMC Ophthalmol. 2007;7: 3 17324285 10.1186/1471-2415-7-3 PMC1831760

[45] Feher J, Kovacs B, Kovacs I, Schveoller M, Papale A, Balacco Gabrieli C. Improvement of visual functions and fundus alterations in early age-related macular degeneration treated with a combination of acetyl-L-carnitine, n-3 fatty acids, and coenzyme Q10. Ophthalmologica. 2005;219(3): 154–166. 15947501 10.1159/000085248

[46] Age-Related Eye Disease Study 2 Research G. Lutein + zeaxanthin and omega-3 fatty acids for age-related macular degeneration: the Age-Related Eye Disease Study 2 (AREDS2) randomized clinical trial. JAMA. 2003;309(19): 2005–2015. 10.1001/jama.2013.4997 23644932

[47] Klein R, Peto T, Bird A, Vannewkirk MR. The epidemiology of age-related macular degeneration. Am J Ophthalmol. 2004;137(3): 486–495. 15013873 10.1016/j.ajo.2003.11.069

[48] Connor KM, SanGiovanni JP, Lofqvist C, Aderman CM, Chen J, Higuchi A, et al Increased dietary intake of omega-3-polyunsaturated fatty acids reduces pathological retinal angiogenesis. Nat Med. 2007;13(7): 868–873. 17589522 10.1038/nm1591 PMC4491412

