# Supplementary material for: Dynamic Drusen Remodelling in Participants of the Nutritional AMD Treatment-2 (NAT-2) Randomized Trial
Source: PLoS One. 2016 Feb 22;11(2):e0149219. doi: 10.1371/journal.pone.0149219 (PMC4762680; doi:10.1371/journal.pone.0149219)
Supplement: S1 Protocol — (DOC) [file pone.0149219.s002.doc]

10, June, 2003

**Double-masked, randomised, parallel, comparative study of oral supplementation with DHA *(Docosahexaenoic acid)*** **versus placebo in the prevention of age related macular degeneration**

##### PROTOCOL n° NAT 2

#### DRAFT N° 6

The information contained in this document is confidential

**INVESTIGATOR**

Dr Eric SOUIED * Tel : 33 1 45 17 59 08

(Service d’ophtalmologie - Hôpital intercommunal de Créteil)

**CO-INVESTIGATORS**

**Ophthalmology**

Pr Gisèle Soubrane * Tel : 33 1 45 17 52 21

(Service d’ophtalmologie - Hôpital intercommunal de Créteil)

**Biology**

Dr Pascale BENLIAN Tel : 33 1 49 28 22 07

Service Biochimie et Biologie moléculaire Fax: 33 1 49 28 22 06

Hôpital Saint-Antoine

184, rue du Faubourg St-Antoine

75 571 PARIS Cedex 12

**STUDY NURSE**

M ……. Antenne du Centre d’Investigation Clinique de l’hôpital H.Mondor.

Hôpital intercommunal de Créteil

*** SITE OF INVESTIGATION**

Service d’Ophtalmologie Tel : 33 1 45 17 59 08

Hôpital intercommunal de Créteil Fax : 33 1 45 17 52 27

40, avenue de Verdun

94 000 Créteil, France

**SPONSOR Contact**

Laboratoire CHAUVIN Study Manager : *Michèle Lablache Combier, PhD*

Clinical Research Clinical study assistant :*Gabrielle BARTHELEMY*

Le Millénaire **Tel**  : 33 1 40 62 35 15 / **Fax** : 33 1 40 62 35 37

BP 1174 Laboratoire CHAUVIN - 18, Avenue de Tourville

F-34009 Montpellier cedex 1 75 007 Paris - France

**PROTOCOL SUMMARY**

| **Name of Company**  Laboratoire CHAUVIN | |
| --- | --- |
| **Study code number**  NAT 2 | |
| **Name of Active Ingredient**  DHA (Docosahexaenoic acid) | |
| **Title of the study**  Double-masked, randomised, parallel, comparative study of oral supplementation with DHA (Docosahexaenoic acid) versus placebo in the prevention of age-related macular degeneration | |
| **Investigator / Coordinator**  Dr Eric SOUIED, Créteil | |
| **Planned study duration**  4 years (3 year follow-up duration for each patient) | **Phase of development**  NA |
| Objectives To evaluate the efficacy and safety of oral supplementation with DHA versus placebo in the prevention of age-related macular degeneration | |
| **Methodology**  Randomised, placebo-controlled, double-masked, parallel, comparative study | |
| **Planned number of patients**  248 | |
| **Diagnosis and main criteria for inclusion**   - Male or female outpatients aged >55 years and < 85 years, - Having given written informed consent, - Presenting neovascular age-related macular degeneration in one eye, - With lesions of age related maculopathy (confluent and diffuse hard drusen or/and soft drusen with or without pigmentary changes or/and reticular pseudodrusen) and with minimal visual acuity of +0.3 LogMAR (≥ 5/10) in the fellow eye (study eye), - Likely to attend the follow-up visits during all the study period.   **Main criteria for exclusion**   - Choroidal new vessels in both eyes, - Wide central area of geographic atrophy encroaching on fovea, in the study eye, - History of other progressive ocular disease which may complicate the assessment of age-related macular degeneration (severe glaucoma, other severe retinopathy..), - Opacity precluding evaluation of retina photographs (cataract, corneal dystrophia..), - History of serious systemic disease which may prevent patient long term participation in the study (mental disorder, cancer, stroke…), - Patients treated with anticoagulants or predisposed to bleeding or haemorrhage, - History of an allergic reaction to fluorescein injection or to indocyanine green or to iodide, - Known sensitivity to DHA or vehicle, - Treatment with Maxepa® or omega-3 polyunsaturated fatty acids within the previous 2 years, - Treatment with alpha-tocopherol acetate (vitamin E), - Any concomitant nutritional supplementation , - Involvement in the last 30 days in any other investigational drug study, - Drug abuse and/or excessive use of medications, - Non-compliant patients (e.g. unlikely to comply with the study protocol), - Monocular patients (for any other reason than age-related macular degeneration). | |

| **Test product, dose and mode of administration**  840 mg daily of DHA : 3 capsules per day; 2 capsules / midday and 1 capsule / evening |
| --- |
| **Duration of the treatment**  3 years for each patient |
| **Reference therapy, dose and mode of administration**  Placebo (olive oil) : 3 capsules per day ; 2 / midday and 1 /evening |
| **CRITERIA FOR EVALUATION**  **Efficacy :** Primary variable Time to occurrence of choroidal new vessels in the study eye  *(The following prognostic factors: age, history of addiction to smoking, total cholesterol and apoE genotype will be considered as well as fish intake frequency)*  **Main Secondary variable(s)**  Percentage of patients for whom new vessels occur during the study and  Change from baseline in:   - Visual acuity (ETDRS) - DHA concentration in red blood cell membrane - Drusen (number, size and area) - Retinal function (multi focal ERG)   **Safety :**   - Slit Lamp Examination results (including grading of lens opacification/LOCS II) - Fasting plasma lipoprotein profile - Adverse or unexpected events |

**TABLE OF CONTENTS**

[1. LIST OF ABBREVIATIONS AND DEFINITION OF TERMS 1](#__RefHeading___Toc43179122)

[2. INTRODUCTION 1](#__RefHeading___Toc43179123)

[3. STUDY OBJECTIVES 4](#__RefHeading___Toc43179124)

[4. INVESTIGATIONAL PLAN 4](#__RefHeading___Toc43179125)

[4.1 Overall study design and plan description 4](#__RefHeading___Toc43179126)

[4.2 Rationale for study design 5](#__RefHeading___Toc43179127)

[4.3 Selection of study population 6](#__RefHeading___Toc43179128)

[4.3.1 Inclusion criteria 6](#__RefHeading___Toc43179129)

[4.3.2 Exclusion criteria 7](#__RefHeading___Toc43179130)

[4.3.3 Removal of patients from therapy or assessment 8](#__RefHeading___Toc43179131)

[5. TREATMENTS 8](#__RefHeading___Toc43179132)

[5.1 Formula and presentation 8](#__RefHeading___Toc43179133)

[5.2 Study drug supply, dispensation, and therapeutic regimen 10](#__RefHeading___Toc43179134)

[5.2.1 Drug supplies 10](#__RefHeading___Toc43179135)

[5.2.2.Drug dispensation 10](#__RefHeading___Toc43179136)

[5.2.3 Therapeutic regimen 11](#__RefHeading___Toc43179137)

[5.3 Compliance with the treatment 11](#__RefHeading___Toc43179138)

[5.4 Randomisation and code-breaking 11](#__RefHeading___Toc43179139)

[5.5 Concomitant therapy 12](#__RefHeading___Toc43179140)

[5.5.1 Permitted therapy 12](#__RefHeading___Toc43179141)

[5.5.2 Prohibited therapy 12](#__RefHeading___Toc43179142)

[6. EFFICACY AND SAFETY VARIABLES 12](#__RefHeading___Toc43179143)

[6.1 Efficacy 12](#__RefHeading___Toc43179144)

[6.1.1 Evaluation criteria 12](#__RefHeading___Toc43179145)

[6.1.2 Examination schedule 13](#__RefHeading___Toc43179146)

[6.1.3 Assessments of efficacy variables 14](#__RefHeading___Toc43179147)

[Primary efficacy variable 14](#__RefHeading___Toc43179148)

[Secondary efficacy variables 15](#__RefHeading___Toc43179149)

[6.2 Safety variables 16](#__RefHeading___Toc43179150)

[6.2.1 Ocular tolerance 16](#__RefHeading___Toc43179151)

[6.2.2 Systemic tolerance 16](#__RefHeading___Toc43179152)

[6.2.3 Adverse or unexpected events 16](#__RefHeading___Toc43179153)

[6.3 Study flow chart 18](#__RefHeading___Toc43179154)

[7. QUALITY ASSURANCE 19](#__RefHeading___Toc43179155)

[7.1 Study monitoring 19](#__RefHeading___Toc43179156)

[7.2 Data recording 19](#__RefHeading___Toc43179157)

[7.3 Archives 19](#__RefHeading___Toc43179158)

[7.4 Auditing / Inspection 20](#__RefHeading___Toc43179159)

[8*.* STATISTICAL ANALYSIS 20](#__RefHeading___Toc43179160)

[8.1 Methods 20](#__RefHeading___Toc43179161)

[8.2 Determination of sample size 24](#__RefHeading___Toc43179162)

[9. PATIENT FOLLOW-UP 25](#__RefHeading___Toc43179163)

[9.1 Protocol deviation 25](#__RefHeading___Toc43179164)

[9.2 Premature withdrawal from the study 25](#__RefHeading___Toc43179165)

[10. ADMINISTRATIVE ASPECTS 25](#__RefHeading___Toc43179166)

[10.1 Duration of the study 25](#__RefHeading___Toc43179167)

[10.2 Confidentiality - Publication 26](#__RefHeading___Toc43179168)

[10.3 Contract 26](#__RefHeading___Toc43179169)

[11. INVESTIGATOR’S APPROVAL AND ETHICAL ASPECTS 26](#__RefHeading___Toc43179170)

[12. REFERENCES 27](#__RefHeading___Toc43179171)

[APPENDICES 29](#__RefHeading___Toc43179172)

[APPENDIX I. DESCRIPTION OF STUDY PROCEDURES 30](#__RefHeading___Toc43179173)

[I.A BEST CORRECTED VISUAL ACUITY 30](#__RefHeading___Toc43179174)

[I.B FUNDUS PHOTOGRAPHY 37](#__RefHeading___Toc43179175)

[I. C DIGITAL FLUORESCEIN AND INDOCYANINE GREEN (ICG) ANGIOGRAPHY 41](#__RefHeading___Toc43179176)

[I. D BIOLOGICAL ANALYSES 42](#__RefHeading___Toc43179177)

[I. E APO E GENOTYPING 43](#__RefHeading___Toc43179178)

[I. F LENS OPACITY CLASSIFICATION SYSTEM (LOCSII) 43](#__RefHeading___Toc43179179)

[I. G MULTIFOCAL ERG. 44](#__RefHeading___Toc43179180)

[APPENDIX II. PATIENT INFORMATION AND CONSENT FORM 45](#__RefHeading___Toc43179181)

[APPENDIX III. ADMINISTRATIVE DOCUMENTS 46](#__RefHeading___Toc43179182)

[APPENDIX IV. JUSTIFICATION OF LAWFUL REGULARITY OF THE STUDIED PRODUCT 47](#__RefHeading___Toc43179183)

# 1. LIST OF ABBREVIATIONS AND DEFINITION OF TERMS

AMD Age-related Macular Degenerarion

CNV Choroidal New Vessel

DHA Docosahexaenoic acid

DNA Deoxyribonucleic Acid

EPA Eicosapentaenoic acid

ERG Electroretinogram

ETDRS Early Treatment Diabetic Retinopathy Study

HDL-C High Density Lipoprotein Cholesterol

ICG Indocyanine Green

LDL-C Low Density Lipoprotein Cholesterol

RBCM Red Blood Cell Membrane

RPE Retinal Pigment Epithelium

S Serum

TC Total Cholesterol

TG Triglycerides

WBC White Blood Cell

# 2. INTRODUCTION

Age related macular degeneration (AMD) is the most common cause of irreversible vision loss in the elderly population in Europe and the United States (1). The common features of AMD are late age of onset and decrease of central vision resulting in central scotoma and legal blindness. The earliest funduscopic finding is that of macular drusen (localized deposits of lipids and lipoproteins underneath the retinal pigment epithelium cells), retinal pigment epithelial clumping, and areas of atrophy. Two forms of AMD are usually described : exudative or atrophic (2, 3). The atrophic form is defined by the presence of degeneration in the central retinal pigment epithelium (RPE), choriocapillaris and photoreceptors resulting from the enlargement and coalescence of small areas of peri-foveolar atrophy. Choroidal neovascularization (CNV) heralds the onset of exudative macular degeneration. Occurrence of CNV is a turning point in the course of the disease, since it induces severe and irreversible complications like serous retinal detachment, edema and hemorrhage, leading to the destruction of the macular photoreceptors. Exudative AMD is responsible for almost 90% of the severe visual loss due to AMD (4). Currently available treatments are limited to laser or photodynamic therapy in some cases of exudative AMD, in order to limit the extension of CNV (3). There is no established reference preventive therapy. Therefore a major challenge facing vision researchers is to find causes and mechanisms of the disease to develop preventive therapies.

To date, etiology of AMD remains unclear. AMD appears to be a multifactorial disease. Several environmental risk factors have been evidenced, including smoking, elevated serum cholesterol levels and systemic hypertension (1). However, a number of studies suggest that AMD has a familial component and that genetic factors contribute to its development. The ε4 allele of the gene coding for apoE (an apolipoprotein involved in the transport of cholesterol and lipids) has been shown to have a protective effect on the occurrence of exudative AMD (soft drusen and choroidal new vessels) (1, 5). These findings evidenced the role of the metabolism of lipids in AMD and leaded us to investigate this pathway. Four recent independent epidemiological studies have shown a significant relationship between high intake of docosahexaenoic acid 22:6 omega 3 (DHA), a long chain poly-unsaturated fatty acid which is predominantly found in oily fish, and a decreased risk of neovascular AMD (6, 7, 8, 9). Furthermore, exudative AMD is less frequent in populations whose nutritional diet is rich in fish and AMD has begun to appear in Iceland or Japan with the shift to a more westernised diet (10). DHA appears to play an important role in the normal development, morphology and function of the retina (11, 12, 13,14). In animals (rats, guinea pig), dietary deficiency in omega 3 fatty acids has been associated with altered function of the retina. Interestingly, impaired vision due to a long period of DHA deprivation remains reversible with a DHA enriched diet. More recently studies in infants, prematured or not, have confirmed the essential role of DHA for the functional development of the retina in human (12 ).

The protective effect of DHA may be related to different mechanisms (15, 16). According to J. Seddon (8), the antithrombotic and hypolipidemic effects of the long chain omega3 fatty acids on the cardio-vascular system could also exert a beneficial effect on the vasculature of the choroids. Several other well known systemic effects of DHA could also play a preventive role in AMD, such as increased high density lipoproteins (HDL), decreased triglycerides (TG), increased apoE, anti-inflammatory role and protective effect on altered tissues such as in myocardium after ischemia reperfusion injury. This last effect, which may imply intracellular regulation as well as plasma membrane remodelling, is assumed to underlay the reduction in sudden death after myocardial infarction seen in large randomized control trials. Moreover, in the Gissi-Prevenzione trial (17, 18), omega 3 fatty acids supplied during 3.5 years reduced significantly (- 20%) overall mortality in myocardial infarction survivors, whom mostly correspond in age and sex to the population of subjects affected by age-related macular degeneration (≥ 55 years old). Finally a recent study comparing omega 6 and omega 3 supplementation in patients with carotid atherosclerosis has shown that indeed omega 3 incorporate into the arterial lesion and stabilize atherosclerotic plaques, in part by reducing its proliferative and inflammatory components (19). Therefore one might expect that once omega 3 fatty acids are incorporated into a human cell membrane they may exert their protective action locally.

Furthermore DHA is known to have specific retinal effects that are likely to play a preventive role in AMD, such as increase mitochondrial activity, increase RPE acid lipase activity, anti-oxydative , anti-proliferative and anti-apoptotic effects (20). DHA represents the main component (> 50%) of the lipids of the external membrane of the photoreceptors cells, suggesting a major role in the maintain of the structure of these cells. On the opposite, an imbalance of the lipidic composition of the retina leads to the degradation of the photoreceptors, and accumulation of deposits of debris made of lipids and lipoproteins, localized at the level of retinal pigment epithelium cells. Since photoreceptor outer segments have a high DHA content and require a constant supply of these omega 3 fatty acids due to their continuous renewal, diets rich in DHA may improve retinal function and protect against the development of exudative AMD (6, 8).

From a safety point of view, the only known side effects of omega 3 fatty acids are digestive intolerance and a modification of biochemical parameters related to platelet aggregation in human consuming very high quantities of omega 3 fatty acids. Numerous studies on omega 3 supplementation performed in the cardiovascular area involved thousands of patients without any significant side effects, particularly no haemorrhagic side effects. In the Gissi-Prevenzione study, 5000 patients aged 50 years or above had no increase in fatal or non-fatal stroke while they received PUFA together with aspirin. The American Food and Drug Administration (FDA) performed a comprehensive evaluation of more than 2600 articles on docosahexaenoic and eicosapentaenoic acids (DHA and EPA), the two long chain poly-unsaturated omega-3 fatty acids found in fish oil. The FDA concluded that dietary intakes of up to 3g daily of DHA and EPA were GRAS [generally recognized as safe] (21).

The safety profile of PUFA has been a strong argument to conduct a study with a nutritional supplement in elderly subjects who are often poly-medicated and more prone to adverse side effects with classical lipid-lowering drugs.

Recently, DHA was given in patients affected with inherited retinitis pigmentosa, an inherited retinal degeneration. A slowing of the retinal degeneration was observed (visual function analyzed by ERG features) compared with the control group. No systemic nor retinal side effect was observed (22). Moreover, a preliminary study called NAT-1 was performed in thirty-eight patients affected with an homogeneous subgroup of AMD without CNV. Among them, 22 patients were orally given DHA at the dose of 480 mg per day in combination with EPA for 6 months. Neither adverse effect nor dropout were observed. It was shown that oral intake of DHA can significantly increase the levels in serum and red blood cell membranes, even in the elderly population (23). It is now interesting to evaluate the potential efficacy of oral supplementation of DHA in preventing the occurrence of new vessels in patients affected with AMD.

# 3. STUDY OBJECTIVES

The objective of the study is to evaluate the efficacy of an oral supplementation with DHA versus placebo in the prevention of the progression of early stages of AMD. The occurrence of choroidal new vessels in AMD patients will be evaluated as a primary criterion while the progression of drusen will be considered as an important secondary criterion.

A secondary purpose is also to confirm the safety of this new treatment.

# 4. INVESTIGATIONAL PLAN

## 4.1 Overall study design and plan description

This is a prospective, single centre, double-blind, randomised, parallel, placebo-controlled, comparative study.

Two hundred and forty eight patients, aged at least 55 and less than 85, presenting neovascular age-related degeneration in one eye and lesions of age related maculopathy (confluent and diffuse hard drusen or /and soft drusen with or without pigmentary changes or/and reticular pseudodrusen) in the second eye (study eye), are planned to be included in one centre.

Randomisation will be balanced for each treatment arm. The total treatment duration for each patient will be 3 years.

Five visits are planned, a screening visit at D0 and four follow up visits at 6 months and at the end of Year 1, Year 2 and Year 3.

This study will bring a direct individual benefit for patients.

Age-related macular degeneration is a leading cause of visual handicap. Its impact on our growing elderly population is rising. Nevertheless, there is no reference treatment to prevent further age-related macular degeneration and therapeutic options are very limited.

Based on results of epidemiological studies in age-related macular disease and based on clinical proofs of its efficacy on cardiovascular or inflammatory diseases, DHA oral supplementation seems to be an attractive and safe preventive therapeutic option for age-related macular degeneration. DHA is likely to delay the occurrence of choroidal new vessels (i.e. occurrence of a severe visual impairment) in patients presenting with early stages of the disease.

Moreover, for all patients, progression of the disease will be regularly evaluated according to the protocol recommended by the AMD specialists (frequency of follow-up visits and performed examinations). This will allow the early detection and required treatment (laser or photodynamic therapy) of choroidal new vessels in order to reduce, as much as possible, the risk for later severe visual loss.

## 4.2 Rationale for study design

AMD is a chronic degenerative disease of the macular region of the retina.

The long treatment duration required in this study (3 years), is suitable for the evaluation of a DHA supplementation as a preventive treatment for AMD. It takes into account the percentage of new vessels occurrence that is around 30 to 36 % in the studied population after 3 years (see section 8.2).

The study design involving two parallel groups is the most suitable for a comparative trial (on an evolutive disease) that requires such a long treatment duration.

Since there is no reference treatment to prevent further AMD it is justified to use a placebo as a comparative treatment. Olive oil is chosen because it is part of a staple diet and has no deleterious effect on the retina.

In the general population the recommended nutritional daily intake of DHA are 120 mg daily.

A preliminary study (NAT 1) has shown that it is possible to significantly increase cell membrane level of DHA in aged population, by an oral intake of 480 mg of DHA (23) .

Since cardiovascular studies did not show any adverse event until the dose of 3g per day of DHA and EPA (21), and knowing that a pharmacological effect is searched in the elderly whose retina is already affected, our purpose is to enhance DHA daily intake and to be close to the 1 g daily level.

Taking into account a number of daily units (3 capsules per day) compatible with a good compliance the chosen DHA daily dose for this study is 840 mg (equivalent to eating 50-100g of fish).

Furthermore, as previously mentioned, the risk for exudative AMD has been associated with polymorphism in the gene encoding apoE : a lower frequency of epsilon 4 allele carriers was found in patients suffering from exudative AMD (5).

Consequently apoE genotype of consenting patients will be evaluated and the efficacy of the treatment with DHA will be assessed considering the apoE genotype.

## 4.3 Selection of study population

### 4.3.1 Inclusion criteria

Eligible patients have to fulfil the following criteria:

1. Male or female outpatients aged ≥ 55 years and < 85 years,
2. Having given written informed consent,
3. Presenting neovascular age-related macular degeneration in one eye,
4. With lesions of age related maculopathy (confluent and diffuse hard drusen or/and soft drusen with or without pigmentary changes or/and reticular pseudodrusen) and with minimal visual acuity of +0.3 LogMAR (≥ 5/10) in the fellow eye (study eye),
5. Likely to attend the follow-up visits during all the study period.

### 4.3.2 Exclusion criteria

Patients who met any of the exclusion criteria below were ineligible for study participation:

1. Choroidal new vessels in both eyes,
2. Wide central area of geographic atrophy encroaching on fovea, in the study eye,
3. History of other progressive ocular disease which may complicate the assessment of age-related macular degeneration (severe glaucoma, other severe retinopathy…),
4. Opacity precluding evaluation of retina photographs (cataract, corneal dystrophia…),
5. History of serious systemic disease which may prevent patient long term participation in the study (mental disorder, cancer, stroke…),
6. Patients treated with anticoagulants or predisposed to bleeding or haemorrhage,
7. History of an allergic reaction to fluorescein injection or to indocyanine green or to iodide,
8. Known sensitivity to DHA or vehicle,
9. Treatment with Maxepa® or omega-3 polyunsaturated fatty acids within the previous 2 years,
10. Treatment with alpha-tocopherol acetate (vitamin E),
11. Any concomitant nutritional supplementation,
12. Involvement in the last 30 days in any other investigational drug study,
13. Drug abuse and/or excessive use of medications,
14. Patients who are unlikely to comply with the study protocol or who are likely to be moving and lost to follow up in the 3 years,
15. Monocular patients (for any other reason than age-related macular degeneration),
16. Patient not covered by the French Social Security scheme,
17. Patient being a ward of court.

### 4.3.3 Removal of patients from therapy or assessment

If a patient did not attend a scheduled control visit, he/she will be contacted again. In case of premature withdrawal from the study, the investigator is to make every effort to determine the reason.

Generally, patients may withdraw or be withdrawn from the study for the following reasons:

- Patient’s decision (in accordance with the Declaration of Helsinki, any patient can voluntarily discontinue the study at any time he or she chooses, without prejudice).
- If the investigator considers that the patient’s health is compromised by remaining in the study.
- If the patient is not sufficiently co-operative.
- If a serious adverse event prevents the patient from attending the follow-up visits.

# 5. TREATMENTS

## 5.1 Formula and presentation

Depending on the randomisation , the patient will receive one of the following treatments :

- Capsules containing 600 mg of fish oil comprising 280 mg of DHA*

| Fish oil  Comprising DHA  and EPA | 600 mg  280 mg  90 mg maximum |
| --- | --- |

*(* see also appendix IV concerning lawful regularity of the product)*

- Capsules of placebo

| Olive oil | 602 mg |
| --- | --- |

Excipient contains trace amount (2 mg) of alpha-tocopherol acetate (vitamin E) as conservative. It contains no allergic substance except for red iron oxide.

Both treatments will have identical presentation in order to preserve masking. Treatments will be supplied as capsules grouped in blister-pack of 20.

Each box will contain 3 blister-packs and so 60 capsules.

3 packages will be provided for each patient, each containing 20 boxes allowing a 1 year treatment.

Each blister-pack will be identified with the following legal mention:

- Sponsor ‘s name
- Study number
- Patient number
- Expiry date
- Use only under medical supervision

Moreover boxes will be labelled in accordance with European GMP, ICH guidelines and local requirements (article R.5123 in France) including:

- Study number

- Investigator’s name

- Patient number - For clinical trial use only

- 3 blister-packs of 20 capsules for oral route - Use only under medical supervision

- directions for use - Keep out of reach of children

- Storage : below 25°C; to shelter from light; - Return any empty packaging

to be kept dry and unused product

- Expiry date

- Sponsor’s name and address

In addition 2 duplicate labels will be provided on each patient package to be stuck on the case report form.

## 5.2 Study drug supply, dispensation, and therapeutic regimen

### 5.2.1 Drug supplies

The investigational products will be dispatched to the hospital pharmacist and delivered to the investigator, packaged and labelled in a manner appropriate to the nature of the trial, as well as in quantities and at the times necessary to enable the investigator to carry out the trial in accordance with the study protocol.

The following products will be supplied:

- Capsules of fish oil including 280mg of DHA: 3600 capsules (i.e 3 packages of 20 boxes, each box containing 60 capsules) per patient.
- Capsules of placebo: 3600 capsules (i.e 3 packages of 20 boxes, each box containing 60 capsules) per patient.

The pharmacist or the investigator will acknowledge receipt of the products supplied. He/she must ensure that they are kept under the appropriate conditions (in a secured location accessible only to study personnel, maintained below 25 °C, sheltered from light and kept dry). At the end of the study, all used and unused study drugs will be fully returned to the sponsor for drug reconciliation.

### 5.2.2.Drug dispensation

1 randomisation list will be established and balanced for treatments. The treatments will be continuously numbered from 001 to 248.

At the end of the baseline evaluation, treatment numbers will be allocated to the patients in ascending order using the next available consecutive number of the list.

The first patient will so receive pack 001 , the second pack 002 and then consecutively.

The double-masked treatment will be dispensed by the investigator or the pharmacist.

3 dispensations will be done during the study:

- First dispensation on D0, at the end of the baseline visit (Visit 1).
- Second dispensation 1 year later, at the end of Visit 3 (Year 1 visit).
- Third dispensation 1 year later, at the end of visit 4 (Year 2 visit).

Each time the patient will receive a package containing 20 boxes of 60 capsules, that is to say 1200 capsules for a 1 year treatment.

Upon each dispensation, the duplicate label provided must be fixed on the corresponding case report form or on a dispensation form.

### 5.2.3 Therapeutic regimen

The patient will take 3 capsules per day: 2 capsules at noon and one capsule in the evening of either DHA (i.e. 840 mg per day : 280mg X 3) or placebo .

The capsules must be swallowed with a glass of water, during the meal.

The duration of the treatment will be 3 years.

## 5.3 Compliance with the treatment

At each visit, the patients have to bring back the empty blister-packs and the unused study medications. The compliance will be recorded in the case report form as yes or no and the frequency of forgotten capsules will be described.

The compliance will be considered to be good when 80% capsules or more are taken by the patient.

At the end of the study, all the study medications will be returned to the sponsor so that drug reconciliation can be established.

Moreover, the DHA level in serum will be a good indicator of patient compliance to treatment.

## 5.4 Randomisation and code-breaking

The randomisation list will be prepared prior to the enrolment using the QL-Ranclin software.

Treatments will be balanced in a 1 to 1 ratio.

At the end of the baseline visit (Day 0), patients who fulfilled all inclusion criteria and none of the exclusion criteria will be randomly assigned to one of the treatment groups (DHA or placebo).

Only in emergencies, and for reasons which must always be given, can the code be broken.

For this purpose sealed opaque envelopes, bearing the number of the patient and containing the name of the allocated treatment, will be given to the investigator or the pharmacist.

It is thus possible to unmask the treatment of one patient only without unmasking all the others.

## 5.5 Concomitant therapy

At each visit, information on any concomitant therapy will be collected and recorded in the case report form.

### 5.5.1 Permitted therapy

Any therapy which does not interfere with the protocol will be allowed including aspirin.

Hypolipidaemic drugs (except Maxepa® and fish oil or DHA containing drugs) are allowed during the study. Their use will be recorded on the CRF and these data will be updated all along the study.

If any CNV have occurred, a suitable therapy (laser therapy or photodynamic therapy) will be performed.

### 5.5.2 Prohibited therapy

Therapies interfering with the protocol are prohibited during the study except if absolutely necessary for the patient’s care:

- Anticoagulants
- Treatment with Maxepa® or omega-3 polyunsaturated fatty acids
- Treatment with alpha-tocopherol acetate
- Any concomitant nutritional supplementation (including supplementation with fish-oil or omega-3 fatty acids and alpha-tocopherol acetate/vitamin E).

# 6. EFFICACY AND SAFETY VARIABLES

## 6.1 Efficacy

### 6.1.1 Evaluation criteria

Primary criterion:

Choroidal neovascularization heralds the onset of exudative macular degeneration, the most severe stage of the disease.

Since this study is designed to evaluate the efficacy of an oral DHA supplementation in the prevention of exudative macular degeneration it is justified to choose the time to occurrence of CNV in the study eye as the main criterion in this study.

The study eye will be the eye not affected with CNV before entering the study.

Secondary criteria:

The ocular criteria will concern systematically the study eye.

- Percentage of patients for whom new vessels occur during the study,
- Change from baseline in visual acuity in LogMar units (ETDRS) and proportion of patients with a visual acuity decrease from baseline of more than 15 letters at ETDRS,
- Change from baseline in visual function (assessed with a multifocal ERG),
- Occurrence and progression of drusen (number, size, and area) ,

all criteria allowing to determine the efficacy of the treatment to slow-down the progression of the disease.

- DHA concentration changes in red blood cell membrane is a surrogate marker for DHA ability to penetrate the retina visual cells.

### 6.1.2 Examination schedule

The different visits will take place on D0, Month 6 (±15 days), Year 1(± 30 days),

Year 2 (±30 days) and Year 3 (± 30 days).

Before inclusion, a baseline assessment will take place on D0. Besides validating the inclusion and exclusion criteria, this baseline visit will also enable the recording of demographic (including ethnicity) and baseline data. Data concerning some identified risk factors for AMD, as age and history of addiction to smocking, will be recorded. Moreover a diet questionnaire must be completed in order to assess the patient’s fish intake frequency.

Any general pathology (particularly diseases predisposing to bleeding or haemorrhage) and ocular pathology as well as any concomitant treatment will be recorded.

These data will be updated all along the study.

For patients previously supplemented with omega-3 fatty acids the corresponding stopping date will be recorded.

On each visit, ophthalmic assessments will include the following:

- Best corrected visual acuity measured with ETDRS charts
- Slit Lamp Examination
- Multifocal ERG (except on 6 month visit)
- Fundus photographs
- Fluorescein angiography (except on 6 month visit)

Moreover a complete profile of blood lipids will be performed on D0, at the 6 month visit and at the 3 year visit : fasting plasma lipoproteins [triglycerides (TG), total cholesterol (TC), HDL-C and LDL-C ] and fatty acids in serum and in red blood cell membranes (RBCM) will be measured. To this end 15 ml of venous blood will be collected after a 12 hour fasting period (as described in appendix I.D).

These data will allow first to evaluate for each patient the compliance to the treatment (DHA level in plasma) and the efficacy of DHA to penetrate the cell membrane (DHA level in RBCM), then to confirm the safety of the treatment concerning the metabolism of lipoproteins (fasting plasma lipoprotein profile).

Moreover since TC is considered as a risk factor for age-related macular degeneration, it will be considered for evaluation of treatment efficacy.

Furthermore, as previously mentioned (see section 4.2), apoE genotype of consenting patients will be evaluated on DNA of white blood cells, on D0.

10 ml of venous blood will be collected. The protocol for extraction of DNA from white blood cells and for evaluation of apoE allele is given in appendix I.E. If appropriate, the efficacy of the treatment will be assessed considering the apoE genotype .

### 6.1.3 Assessments of efficacy variables

### Primary efficacy variable

The occurrence of choroidal new vessels in the study eye will be determined from prospective assessment of fluorescein angiography every year.

Patients will be asked to report any new visual symptoms referable to the study eye between visits and to come back immediately for an angiography. Then a suitable laser or photodynamic therapy will be performed if any CNV have occurred.

Fluorescein angiography will allow to detect the presence and specify the location (extrafoveal, juxtafoveal, subfoveal), type (classic, occult) of CNV. It will be performed, through the dilated pupil, after intra-venous injection of 10% sodium fluorescein. This examination will be performed first on D0 for both eyes, then yearly.

If CNV are suspected in the study eye, an indocyanine green (ICG) angiography will be also performed to confirm their occurrence and characterize them precisely.

The protocol for fluorescein and ICG angiography is described in appendix I.C.

### Secondary efficacy variables

Fundus photographs (red free frame), performed through the dilated pupil, will allow :

- first to describe the type, number, size and area of drusen on D0,
- then to evaluate the occurrence and progession of drusen (number, size and area), in the study eye, at each follow-up visit.

For this purpose, an automatic quantification of drusen will be performed by LERISS laboratory using an image enhancement and automatic detection and quantification of drusen software. The protocol for quantification and evaluation of evolution of drusen is described in appendix I. B.

Change in visual acuity will be evaluated from baseline using the ETDRS charts. Best corrected visual acuity will be evaluated, before pupil dilatation, by the same person at each visit. The protocol for evaluation of visual acuity is given in appendix I. A.

A significant decrease in visual acuity is defined as a decrease of more than 15 letters at ETDRS.

Multifocal ERG modification from baseline will be evaluated yearly on the study eye. ERG responses will be obtained from the anesthetized cornea, through the dilated pupil, with a bipolar contact lens electrode (see appendix I.G). ERG must be performed before fundus photographs and angiography.

DHA concentration in red blood cell membranes (RBCM) will be performed on D0, at the 6 month visit and at the 3 year visit. For this purpose, 5 ml of 12 hour fasting blood samples will be taken in the early morning. DHA concentration change in red blood cell membrane is considered as the witness of the DHA ability to penetrate the retina visual cells*.* The protocol for dosage of fatty acids in RBCM is given in appendix I.D.

## 6.2 Safety variables

### 6.2.1 Ocular tolerance

Objective ocular tolerance will be assessed in each eye at each visit, by an examination of the eye with the slit lamp. This examination will be performed without fluorescein or dye on each visit.

Slit lamp examination results will be recorded as normal or abnormal. Abnormalities will be described in the case report form. If any abnormality is noticed which was either not present at baseline or which is worse than at baseline, then the investigator will have to complete an adverse event form .

A precise evaluation of the lens opacity will be performed through the dilated pupil, using the LOCS II scale (appendix I.F).

### 6.2.2 Systemic tolerance

Fasting plasma lipoproteins profile [triglycerides (TG), total cholesterol (TC), HDL-C and

LDL-C] will be performed on D0, at the 6 month visit and at the 3 year visit. To this end blood samples after fasting will be taken. The protocol for dosage is described in appendix I.D. If any abnormality is noticed which was either not present at baseline or which was worse than at baseline, then the investigator will have to complete an adverse event form .

Moreover, all systemic adverse events, particularly digestive adverse events (nausea, eructations, diarrhea or abdominal pain) and bleeding events*, will be registered all along the study (see paragraph 6.2.3).

** because there is no simple, specific and accurate biologic index allowing to predict the risk for bleeding.*

### 6.2.3 Adverse or unexpected events

Adverse or unexpected event occurring during the study (from the time of patient inclusion to that of withdrawal or completion ) should be reported regardless of whether the event is attributed or not to the study medication.

The onset of any event is recorded on the case report form at each visit. The complete description and characteristic of each event is recorded in the adverse events section at the end of the case report form.

In order to avoid bias in the patient’s answers, general questions with no hint of suggestion must be used.

If an event is serious, a complementary report of a serious adverse event form (last page of case report form) must be completed and transmitted to the sponsor as soon as possible.

The sponsor and the Study Manager or the Clinical Research Assistant are to be notified of any serious adverse event by telephone (N° 01 40 62 35 35 or 01 40 62 35 15) or by fax (N° 01 40 62 35 37) or by e-mail (michele.lablache-combier@bausch.com).

A serious adverse event or serious adverse drug reaction is defined by ICH guidelines as "Any untoward medical occurrence that at any dose : results in death, is life-threatening, requires inpatient hospitalisation or prolongation of existing hospitalisation, results in persistent or significant disability/incapacity, or is a congenital /birth defect".

The sponsor is responsible for the prompt notification of serious adverse effects to the Ethics Committee. The investigator shall supply the sponsor and the Ethics Committee with any additional requested information, notably for reported death of a subject.

If the name of the study treatment must be known in order to treat a serious reaction, the code can be broken according to the procedure defined in section 5.4.

## 6.3 Study flow chart

| | **STUDY PROCEDURE** | **Visit 1** | **Visit 2** | **Visit 3** | **Visit 4** | **Visit 5** | | --- | --- | --- | --- | --- | --- | | **Baseline** | **6 months*** | **1 year**** | **2 years**** | **3 years**** | | Informed written consent | **X** |  |  |  |  | | Demographic data | **X** |  |  |  |  | | Medical history- Concomitant pathologies and treatment | **X** |  |  |  |  | | Inclusion criteria | **X** |  |  |  |  | | Diet questionnaire | **X** |  |  |  |  | | Blood samples *****  *12h fasting* | **X**  25 ml | **X**  15 ml |  |  | **X**  15 ml | | Visual acuity (ETDRS) | **X** | **X** | **X** | **X** | **X** | | Slit Lamp Examination | **X** | **X** | **X** | **X** | **X** | | Multifocal ERG ***** | **X** |  | **X** | **X** | **X** | | Fundus Photographs | **X** | **X** | **X** | **X** | **X** | | Fluorescein angiography  (+/-ICG angiography) | **X** |  | **X** | **X** | **X** | | Adverse event report |  | **X** | **X** | **X** | **X** | |
| --- | --- | --- | --- | --- | --- | --- | --- | --- | --- | --- | --- | --- | --- | --- | --- | --- | --- | --- | --- | --- | --- | --- | --- | --- | --- | --- | --- | --- | --- | --- | --- | --- | --- | --- | --- | --- | --- | --- | --- | --- | --- | --- | --- | --- | --- | --- | --- | --- | --- | --- | --- | --- | --- | --- | --- | --- | --- | --- | --- | --- | --- | --- | --- | --- | --- | --- | --- | --- | --- | --- | --- | --- | --- | --- | --- | --- | --- | --- | --- | --- | --- | --- | --- |

***** not part of usual follow-up for AMD * Schedule ± 15 days

** Schedule ± 30 days

# 7. QUALITY ASSURANCE

## 7.1 Study monitoring

Periodically a study promotor representative will contact the investigator and the study nurse* and will visit the centre. He/she will ensure that the study is progressing in compliance with the protocol, the local regulations and Good Clinical Practices.

The investigator will make the relevant clinical files available to the clinical study assistant who will maintain patient’s anonymity*.*

** the study is conducted in this centre in partnership with the CIC (Centre d’Investigation Clinique) of Créteil : a CIC study nurse will coordinate the study logistic in the centre.*

## 7.2 Data recording

The investigator is to ensure that all data are recorded on the case report forms as measurements are taken and examinations performed. Data are recorded by writing directly on the case report form or by transcribing from the patient’s original file.

The minimum data recorded in the patient’s original file will include: patient’s history and concomitant medication, patient’s inclusion in the study, all visit dates, summary of clinical condition with adverse events, date of study end or premature withdrawal with reason.

Each page of the case report form must be identified by the patient’s number and the investigator ‘s (or his/her representative’s ) signature (or initials).

Any correction must be dated and countersigned. Data are to be recorded with a ball-point pen.

## 7.3 Archives

Each page of the case report form is in triplicate, one is for the investigator’s archiving file and the other 2 are for the sponsor.

Likewise, the patient consent form is in triplicate. The first copy is kept by the investigator in the archive file, the second is kept by the sponsor in a sealed opaque envelope to maintain patient’s anonymity and the third copy is given to the patient.

All the study documents [list of patients included in the study, patient consents, case report forms and source documentation (including printed fundus photograps and digital images files saved on floppy or CD rom), signed protocol, investigator’s brochure and correspondence] must be archived according to ICH Guidelines.

## 7.4 Auditing / Inspection

During or after the study, the sponsor may carry out an audit of this clinical study. An auditor will be appointed by the sponsor. There may be regulatory inspection(s) and/or IEC reviews. All or part of the study related documents or source data will be verified to ensure that the standards of ICH Good Clinical Practices are met.

The investigator agrees beforehand to provide all the necessary documents and information for the audit (or inspection) to be performed.

# 8*.* STATISTICAL ANALYSIS

## 8.1 Methods

Analysis population

Three populations will be defined for the purposes of analysis.

Safety population

This comprises all patients randomised who had taken at least one unit of study medication.

Intent-to-treat (ITT) population

This comprises all patients in the safety population . The full analysis set arising from this population will comprise all available data from these patients, thus patients for whom there is no post-baseline data will not contribute to this data set.

Per-protocol (PP) population

This comprises all patients in the intent-to-treat population who did not deviate from the protocol in any way likely to seriously affect the primary outcome of the study.

The patients in each analysis population will be agreed on blind as to the treatment received and before statistical analysis begins.

The analysis of the primary efficacy variable will be performed on both the intent-to-treat and per-protocol populations.

The objective of the trial is to show the superiority of DHA versus placebo in preventing the occurrence of new vessels in patients with AMD.

STATISTICAL METHODS

All analyses will be carried out using SAS version 8.2 for PC. Tables and listings will be output directly for use in Word.

All data will be listed in individual patient listings.

Missing data will not be extrapolated.

Efficacy variables

The ocular criteria will concern systematically the study eye i.e. the eye not affected with CNV before entering the study.

The primary efficacy criterion is the time to occurrence of new vessels.

Secondary efficacy criteria:

- Percentage of patients for whom new vessels occur during the study,
- Change from baseline in visual acuity in LogMar units (ETDRS) and proportion of patients with a visual acuity decrease from baseline of more than 15 letters at ETDRS,
- Change from baseline in visual function (assessed with a multifocal ERG),
- occurrence and progression of drusen (number, size and area),
- DHA concentration increase in red blood cells membrane

Safety variables

The ocular criteria will concern both eyes.

- Slit Lamp Examination

- Profile of plasma lipoproteins

- Adverse or unexpected events

Other variables

Other variables recorded consist of demographic and baseline variables as well as history.

Concomitant medication, both systemic and ocular will be recorded.

Compliance to study drugs will be recorded as “yes” or “no” at each visit.

Extent of exposure will be calculated as the number of days on which the study medication has been taken.

Methods of analysis

Primary Efficacy Variable

The primary outcome of the study will be the time to occurrence of CNV in the study eye.

The occurrence rates in the 2 treatment groups will be compared using life-table methods.

The data will be analysed on an intent-to-treat principle so that data on time to occurrence of CNV is collected for all patients where possible, including early withdrawals from medication.

The difference between treatments will be tested using a log-rank test.

Prognostic factors which may affect the outcome, namely age, history of addiction to smoking, fish intake frequency, total cholesterol (TC) and APOE genotype will be considered for inclusion in the model.

Significance of these covariates, either time-dependent or time-independent, will be evaluated by proportional hazard model, the efficacy of the treatment will be adjusted by these covariates if necessary.

Data from patients withdrawn early will be considered censored at the point of withdrawal.

Secondary efficacy variables

The percentage of patients for whom CNV occur during the study will be compared between groups using a CHI square test or a Fisher test when required.

The change from baseline (time of enrollment ) in visual acuity scores at 6 months,Year 1, Year 2 and Year 3 will be compared between treatment groups by means of a Student t-test or a Wilcoxon Rank Sum test depending on data normality. The proportion of patients with a visual acuity decrease from baseline of more than 15 letters on ETDRS charts will be also compared across treatment groups using CHI square test or Fisher test.

The change from baseline in visual function (multifocal ERG),e.g change in amplitude and in timing, at Year 1, Year 2 and Year 3, will be compared between groups by means of a Student t-test or a Wilcoxon Rank sum test.

The progression of drusen (change from baseline in number, size and area) at 6 months, Year 1, Year 2 and year 3 will be compared between groups using a Wilcoxon Rank sum test .

The DHA concentration increase in red blood cells membrane at 6 months and at 3 years will be compared across groups using a Student t-test or a Wilcoxon test depending on data normality.

Safety variables

At each follow-up visit, the percentage of patients with changes (from the previous assessment) in the slit lamp examination will be compared between groups by means of a CHI square test or a Fisher test .

Change from baseline in profile of plasma lipoproteins will be compared between groups using a Student t-test or a Wilcoxon Rank Sum test depending on data normality.

Adverse or unexpected events will be counted and described by visit. In case of a sufficient number of adverse events, the percentage of patients having at least one adverse event will be compared between the 2 groups by means of a CHI square test. In case of serious adverse events, these will be counted and described completely and the corresponding patient profile will be detailed.

Other variables

The comparability of quantitative parameters between groups on D0 will be analysed by a Student t-test or a Wilcoxon Rank sum test depending on data normality. Qualitative parameters will be analysed by a CHI square test or a Fisher test depending on the number of patients in classes.

## 8.2 Determination of sample size

The primary criterion is the time to occurrence of choroidal new vessels (CNV) in the study eye.

The occurrence rates of CNV in both treatment groups will be compared using a log-rank test.

According to previous studies (24) the 3 year risk of developing CNV in one eye is about 30 % (10% per year) for patients with CNV in the fellow eye.

Based upon expectation that DHA-treatment should reduce this rate to 15% at 3 years after D0 , sample size was thus performed assuming a 3 year CNV risk for the study eye in the treated group and in the placebo group to be 15% and 30% respectively.

The number of patients has been calculated with the Pass software for survival curves according to the following hypotheses:

- 2 tailed test
- Type I error α = 0.05
- Type II error β = 0.20 (80% power)
- Regular recruitment and Inclusion period: 1 year
- Follow up period per patient : 3 years
- Equal proportion of patients in each treatment group
- Drop-out rate in each group : 20%

Based on the above specifications the total number of patients should be 245.

Data on 248 evaluable patients are to be available (124 per group).

# 9. PATIENT FOLLOW-UP

## 9.1 Protocol deviation

The reason for deviations (inclusion errors, failure to comply with protocol ...), will be specified in all cases.

Assessments will continue until the end of the study unless the patient's condition requires to discontinue the study.

## 9.2 Premature withdrawal from the study

If a patient does not attend the scheduled control visit, he/she should be contacted again. In case of withdrawal, the reasons will be sought.

Generally, patients may withdraw or be withdrawn from the study for the following reasons:

- of their own free will
- if the investigator considers that the patient's health is compromised by remaining in the study
- if the patient is not sufficiently co-operative
- if a serious adverse event prevents the patient from attending the follow-up visits.

For any premature withdrawal, the investigator must give the date and the reason, and if possible provide an assessment of the therapeutic response.

# 10. ADMINISTRATIVE ASPECTS

## 10.1 Duration of the study

The inclusion period will be 12 months.

The scheduled study duration for each patient will be 3 years.

So the total duration of the study will be 4 years.

## 10.2 Confidentiality - Publication

All information concerning the product and the study is considered to be confidential and should not be disclosed to persons not involved in the study.

The results may only be published after mutual agreement between the sponsor and the investigator.

## 10.3 Contract

A contract will be drawn between the investigator and the sponsor stating the legal and financial aspects involved throughout the study.

# 11. INVESTIGATOR’S APPROVAL AND ETHICAL ASPECTS

The study protocol, patient’s information and consent forms will be submitted to the appropriate Ethics Committee (CCPPRB). The study will start only after approval is obtained.

The clinical study will be conducted in compliance with local regulations, ICH Good Clinical Practices, ICH guidelines and the Declaration of Helsinki.

Freely given, free informed written consent must be obtained from the patients and countersigned by the investigator. Written informed consent will be obtained from each patient as a condition of enrolment in the study. The patient may also participate in the genetic study after obtaining his / her written consent for participation in this research. Consent will be obtained after providing the prospective study participants with an adequate explanation of the protocol procedures and prior to the patient’s participation in the study (including screening procedures).

The undersigned investigator, has read this protocol and considers that it contains all the necessary information to conduct the study.

Signature of CHAUVIN representative Investigator’s Signature

Name Name

Date Date

# 12. REFERENCES

1. Souied E, Kaplan J, Coscas G, Soubrane G. DMLA et génétique. J Fr Ophthalmol 2001 ; 24, 8 : 875-885.
2. Soubrane G, Coscas G. Dégénérescence maculaire liée à l’âge in : Encycl Méd Chir (Elsevier, Paris), Ophtalmologie, 21-249-A-20, 1998, 29p.
3. Soubrane G, Haddad W, Coscas G. Age related macular degeneration. Presse Med 2002 Aug 24; 31 (27) : 1282-1287.
4. Hyman L, Neborsky R. Risk factors for age-related macular degeneration : an update. Current opinion in Ophthalmology 2002; 13 : 171-175.
5. Souied EH, Benlian P, Amouyel P et al. The epsilon 4 allele of the apolipoprotein E gene as a potential protective factor for exudative age-related macular degeneration. Am J Ophthalmol 1998; 125 : 353-359.
6. Smith W, Mitchell P, Leeder SR. Dietary fat and fish intake and age-related maculopathy. 2000; 118 : 401-404.
7. Cho E, Willett WC, Spiegelman D, Rimm EB, Seddon JM, Colditz GA, Mankinson SE. Prospective study of dietary fat and the risk of age-related macular degeneration. Am J Clin Nutr 2001; 73 : 209-218.
8. Seddon JM, Rosner B, Sperduto RD, Yannuzzi L, Haller JA, Blair NP, Willett W. Dietary fat and risk for advanced age-related macular degeneration. Arch Ophtalmol 2001 (Aug); 119(8) : 1191-1199.
9. San Giovanni JP, Chandra SR, Chew EY, Friberg TR, Klein ML, Kuriny N, Seddon JM. Dietary omega-3 long chain polyunsaturated fatty acids and risk for age-related macular degeneration in: ARVO annual meeting. 2003. Fort Lauderdale. Abstract paper 2112.
10. Richer S. Part I : A protocol for the evaluation and treatment of atrophic age-related macular degeneration. J AM Optom Assoc 1999; 70 : 13-23.
11. Neuringer M, Anderson GJ, Connor WE. The essentiality of N-3 fatty acids for the development and function of the retina and brain. Ann Rev Nutr 1988; 8 : 517-541.
12. Simopoulos AP. Omega-3 fatty acids in health and disease and in growth and development. Am J Clin Nutr 1991; 54 : 438-463.
13. Robinson WG, Kuwabara T, Bieiri JG. The roles of vitamin E and unsaturated fatty acids in the visual process. Retina 1982; 2 : 263-281.
14. Bazan NG.The metabolism of omega-3 polyunsaturated fatty acids in the eye: the poss ible role of docosahexaenoic acid and docosanoids in retinal physiology and ocular pathology. Prog Clin Biol Res 1989; 312 : 95-112.
15. Grundy S. N-3 fatty acids. Priority for post-myocardial infarction clinical trials. Circulation 2003; 107 : 1834-1836.
16. Das U. Beneficial effect(s) of n-3 fatty acids in cardiovascular diseases: but, why and how? Prostaglandins, leukotrienes and essential fatty acids 2000; 63(6) : 351-362.
17. Gissi-Prevenzione investigators. Dietary supplementation with n-3 polyunsaturated fatty acids and vitamin E after myocardial infarction: results of the Gissi-prevenzione trial. The Lancet 1999; 354 : 447-455.
18. Marchioli R, Schweiger C, Tavazzi L, Valagussa F. Efficacy of n-3 polyunsaturated fatty acids after myocardial infarction: results of Gissi-Prevenzione trial. Lipids 2001; 36 (supplement) : 5119-5125;
19. Thies F, Garry J, Yaqoob P, Rerkasem K, Williams J, Shearman C, Gallagher P, Calder P, Grimble R. Association of n-3 polyunsaturated fatty acids with stability of atherosclerotic plaques: a randomized controlled trial. The Lancet 2003; 361 : 477-485.
20. Rotstein NP, Politi LE, German OL, Girotti R. Protective effect of docosahexaenoic acid on oxidative stress-induced apoptosis of retina photoreceptors. Invest Ophthalmol Vis Sci 2003; 44(5) : 2252-2259.
21. Substances affirmed as generally recognized as safe : menhaden oil. Fed Regist. June 5, 1997; 62 : 30751-30757.
22. Hoffman DR, Wheaton DH, Locke KG, Fish GE, Birch DG. Four-year outcomes from a randomized clinical trial of docosahexaenoic acid (DHA) supplementation in X-linked retinitis pigmentosa (XRLP) in: ARVO annual meeting. 2003. Fort Lauderdale. Abstract poster 4851/B510.
23. Souied E, Benlian P, Chanu B, Roquet W, Coscas G, Soubrane G. NAT1: A feasibility study of oral DHA supplementation as a nutritional AMD treatment in: ARVO annual meeting. 2003. Fort Lauderdale. Abstract poster 4994/B653.
24. Coscas G. Dégénérescences maculaires acquises liées à l’âge et néovaissseaux sous-rétiniens. SFO report 1991 (Masson, Paris) : 160-161.

# APPENDICES

**APPENDIX I. DESCRIPTION OF STUDY PROCEDURES**

A BEST CORRECTED VISUAL ACUITY

B FUNDUS PHOTOGRAPHY

C FLUORESCEIN AND INDOCYANINE GREEN ANGIOGRAPHY

D BIOLOGICAL ANALYSES

E APO E GENOTYPING

F LENS OPACITY CLASSIFICATION SYSTEM (LOCS II)

G MULTIFOCAL ERG

**APPENDIX II. PATIENT INFORMATION AND CONSENT FORMS**

GENERAL STUDY

GENETIC STUDY

**APPENDIX III. ADMINISTRATIVE DOCUMENTS**

APPROVAL OF THE CCPPRB

INSURANCE CERTIFICATE

**APPENDIX IV. JUSTIFICATION OF LAWFUL REGULARITY OF THE STUDIED PRODUCT**

# APPENDIX I. DESCRIPTION OF STUDY PROCEDURES

### I.A BEST CORRECTED VISUAL ACUITY

**(adapted from AREDS Manual of Operations)**

1. Visual Acuity Equipment and Facilities
   1. Introduction. The visual acuity of participants will be measured according to the standard procedure developed for the Early Treatment Diabetic Retinopathy Study (ETDRS) and adapted for the Age-Related Eye Disease Study (AREDS). The procedure is described in this section. The following equipment is used: a set of three Lighthouse Distance Visual Acuity Test charts (second edition), which are modified ETDRS Charts 1, 2, and R, and a retroilluminated box providing standardized chart illumination, as modified from the design by Ferris and Sperduto. The charts and boxes are manufactured by:

Lighthouse Low Vision Products

36-02 Northern Boulevard

Long Island, New York 11101

Telephone: + 1 718-937-6959

**Visual acuity testing is required at a distance of 4 meters and, for participants with sufficiently reduced vision, at 1 meter. The 4­-meter distance should be marked clearly and permanently; the 1-meter distance must be measured, with a 1-meter stick, with the participant in a chair (Section 1.5).**

- 1. Visual acuity charts. Charts 1 and 2 are used for testing the right and left eye, respectively, and Chart R is used for refraction. The features of the charts are five high-contrast Sloan letters in each of 14 lines, lines of equal difficulty, and a geometric progression of letter size (and, thus, an arithmetic progression of the logarithm of minimum angle of resolution) from line to line. Charts 1, 2, and R have different letter sequences. Participants should be prevented from seeing Charts 1 and 2 until refraction has been completed and the visual acuity test begins.

1.3 Visual acuity box. The dimensions of the light box are 24 and 3/4 inches by 25 and 3/4 inches by 7 inches. The box can be mounted on a wall or on a cylindrical stand manufactured by Lighthouse Low Vision Products. The stand is mounted on a five-pronged wheel base, with each prong about l4 inches long; two of the five wheels are lockable. When the box is mounted on the stand, its height can be varied.

The light box should be mounted at a height such that the top of the third row of letters (0.8 logMAR) is 49 ± 2 inches from the floor.

The rear of the box provides storage space for the two charts not being used.

1.4 Illumination. Most of the room lights should be turned off during the visual acuity test. The box itself provides sufficient illumination for the examiner to record the test results. Additional light can have an adverse effect. With the box light off, not more than 15 footcandles of light should fall on the center of the chart.

The visual acuity light box is equipped with two General Electric Cool Daylight 20-watt fluorescent tubes and a ballast. Because the illumination of fluorescent tubes diminishes by 5 percent during the first 100 hours and by another 5 percent during the next 2,000 hours,

- New tubes should be kept "on" for about 4 days (96 hours, does not have to be continuous), and
- All tubes should be replaced once a year.

The fluorescent tubes should also be checked periodically for proper functioning. Replacement tubes can be purchased at a local store or from Lighthouse Low Vision Products.

Each tube is partly covered by a 14-inch fenestrated sleeve, open in the back, which serves as a baffle to reduce illumination. Each sleeve should be centered on the tube such that an equal length of tube (4 and 3/16 inches) is left uncovered to the right and left of the sleeve. The openings in the backs of the sleeves should be oriented to point directly toward the back of the box (i.e., the sleeves should not be tilted up or down). Also, the lower sleeve has a cutout that should point down toward the ballast.

1.5 Four- and 1-meter visual acuity lanes. A distance of exactly 4 meters (13 feet and 1.5 inches, or 157.5 inches) is required between the participant's eyes and the visual acuity chart for the 4-meter test, and a distance of exactly 1 meter (39 and 3/8 inches) is required for the 1-meter test.

The room for visual acuity testing must have, in addition to the 4-­meter lane, space for the visual acuity box (and possibly a stand) and space for the participant. Minimum room-length requirements vary according to how the box is mounted and whether the participant sits in a chair or stands for the 4-meter test.

- Wall-mounted box: In addition to the 4-meter lane, 7 inches must be allowed for the depth of the box plus space for the participant to sit or stand.
- Stand-mounted box: In addition to the 4-meter lane, 13 inches must be allowed for two of the stand's casters to touch the rear wall (or a line marked on the floor when there is no wall) plus space for the participant to sit or stand.

Marking the distance

4 meters

1. If the chair and visual acuity box are permanently affixed, distance measurements need to be made only once and no floor marks are needed to ensure the correct distance.
2. If the box is mounted on the wall but the participant's chair is not permanently affixed, the 4-meter distance of the participant's eye from the chart must be marked clearly and permanently.
3. If the box is mounted on a movable stand, the 4-meter distance must be marked clearly and permanently on the floor. The location and orientation of the box must be rechecked each time a new chart is put in place or the box is touched. When the stand touches the rear wall of the room, two of the five casters should touch the wall.

1 meter

The 1-meter distance is measured from the eye of the participant, seated comfortably in a chair with his or her back firmly placed against the chair's back, to the center of the second or fourth letter of the third line of the chart, using a 1-meter stick. The stick may be homemade (e.g., a dowel rod) or purchased at a local hardware store or by mail (e.g., from Johnson Level and Tool Manufacturing Company, Inc., Mequon, Wisconsin).

2. Testing Best-Corrected Visual Acuity

2.1 **Four-meter test**. **TESTING OF ALL EYES BEGINS AT 4 METERS**. First, the right eye is tested with Chart 1 and then the left eye is tested with Chart 2. Each chart should remain hidden from view until the eye in question is ready for testing.

The distance from the participant's eyes to the visual acuity chart must be exactly 4.0 meters (13 feet and 1.5 inches, or 157.5 inches). The participant may stand or sit for the 4-meter visual acuity test. If the participant is seated, his or her back should fit firmly against the back of the chair. The examiner should ensure that the participant is standing or sitting comfortably, that the head does not move forward or backward during the test, and that the participant's eyes remain at the 4-meter distance.

The testing procedure for visual acuity is based on the principle that the objective is to test visual acuity and not intelligence or the ability to concentrate or follow or remember instructions (although all of these factors are involved). The participant should be told that the chart has letters only and no numbers. If the participant forgets this instruction and reads a number, he or she should be reminded that the chart contains no numbers and the examiner should request a letter in lieu of the number.

The participant should be asked to read slowly (at a rate not faster than about one letter per second) in order to achieve the best identification of each letter and to not proceed until the participant has given a definite response. It may be useful for the examiner to demonstrate the letter-a-second pace by reciting "A, B, C, . . . ." If, at any point, the participant reads quickly, he or she should be asked to stop and read slowly. If the participant loses his or her place in reading or the examiner loses his or her place (possibly because the letters are read too quickly), the examiner should ask the participant to go back to where the place was lost. Examiners should never point to the chart or to specific letters on the chart or read any of the letters during the test.

Each letter is scored as right or wrong (Section 2.3). Once a participant has identified a letter with a definite single-letter response and has read the next letter, a correction of the previous letter cannot be accepted. If the participant changes a response aloud (e.g., "That was a 'C,’ not an 'O’.”) before he or she has read aloud the next letter, then the change should be accepted. If the participant changes a response after beginning to read the next letter, the change is not accepted.

When the participant says he or she cannot read a letter, he or she should be encouraged to guess. If the participant identifies a letter as one of two or more letters, he or she should be asked to choose one letter and, if necessary, to guess even if the next letter has already been read. The examiner may suggest that the participant turn or shake his or her head in any manner if this improves visual acuity. If the participant does this, care must be taken to ensure that the fellow eye remains covered. When it becomes evident that no further meaningful readings can be made, despite urgings to read or guess, the examiner should stop the test for that eye.

There are several reasons for encouraging participants to guess: (1) participants' statements that they cannot identify a letter are often unreliable; (2) encouraging them to guess helps to maximize the participant's effort; (3) it helps to assure

uniformity among procedures performed by different investigators.

2.2 **One-meter test**. Eyes reading 19 or fewer letters correctly at 4 meters should be tested at 1 meter. If the trial frame is to be removed when changing the test distance from 4 meters to 1 meter, the testing chart (Chart 1 or 2) should first be removed from view to prevent the participant from reading the chart with the fellow eye.

Before testing at 1 meter, a +0.75 sphere should be added to the 4­-meter correction already in the trial frame to compensate for the closer testing distance. The participant may stand or sit for the 4-meter test, but must sit for the 1-meter test. (As indicated in Sections 1.5 and 3.1, the participant should be seated comfortably with his or her back firmly placed against the back of the chair.) The avoidance of any head movement forward or backward is particularly important during the 1-meter test. The participant should be asked to read only the first six lines at 1 meter, making 30 the maximum score attainable at that distance (Section 2.3).

After the test of the right eye is completed, occlude the right eye and replace Chart 1 by Chart 2. The test is repeated for the left eye, starting at 4 meters. When testing of the left eye is completed, Chart 2 should be removed from view; Chart R may be mounted in preparation for the next participant.

2.3 Scoring best-corrected visual acuity. The examiner records each letter identified correctly by circling the corresponding letter on the Visual Acuity Worksheet. Letters read incorrectly and letters for which no guesses are made are not marked on the form. Each letter read correctly is scored as, one point. The score for each line (which is zero if no letters are read correctly) and the total score for each eye are recorded on the Visual Acuity Worksheet after testing is completed. If testing at 1 meter is not required, 30 points are automatically scored for the 1-meter test. The total combined score (i.e., the sum of the 4- and 1-meter scores) and the approximate Snellen fraction, which is determined based on the lowest line read, are recorded on the Visual Acuity Worksheet and on the appropriate Case Report Form.

Exhibit 1, taken from Ferris et al, lists visual acuity in various units of measurement equivalent to the acuity represented by the expected number of letters identified correctly on the ETDRS or Lighthouse charts.

- 1. Light Perception and no light perception. If visual acuity is so poor that the participant cannot read any of the largest letters at 1 meter (i.e., the number of letters read correctly at 1 meter is zero), light perception should be tested with an indirect ophthalmoscope in a darkened room. The indirect ophthalmoscope light

should be in focus at 3 feet with the rheostat set at maximum voltage. From a distance of 3 feet, the beam should be directed in and out of the eye at least four times, and the participant should be asked to respond when he or she sees the light. If the examiner is convinced that the participant perceives the light, vision should be recorded as "light perception"; if not, vision should be recorded as "no light perception."

- 1. Legal blindness. Assessing legal blindness (20/200 or worse) with the visual acuity charts used in this study may present a problem. On standard Snellen charts, the line below 20/200 is 20/100, and so the usual definition of legal blindness (20/200 or worse) could be reworded "worse than 20/100.” The ETDRS charts, however, contain two lines, 20/160 and 20/125, between the 20/200 and 20/100 lines, and so a participant, who should be considered legally blind, may actually read better than 20/200 but worse than 20/100 when tested on the ETDRS charts. This may prevent participants from being designated legally blind, depriving them of economic and social benefits. It is, therefore, suggested that legal blindness be assessed with standard Snellen acuity charts.

**Exhibit 1. ETDRS CHART EQUIVALENT VISUAL ACUITY MEASUREMENTS**

In order to use this ETDRS Chart Equivalent Visual Acuity Measurement Table, it is necessary to obtain the Visual Acuity Score (sum of lines A, B and C on the worksheet). For example if the Visual Acuity Score is 65 letters, the logMAR units would be 0.4. The formula for the conversion is as follows:

logMAR units = 1.7 – (Visual Acuity Score*0.02)

1. **Visual Acuity ( on following page)**

CIRCLE each letter the participant identifies correctly and write the total correct for each row in the column at the right. If the total number of letters read correctly is less than 20, move the participant to a distance of 1 meter from the chart and test the acuity at this distance using only the first six rows of test letters. Prior to actual testing at 1 meter, add a +0.75 sphere to the distance correction in the trial frame.

### I.B FUNDUS PHOTOGRAPHY

***Photographer Experience***

Experimented photographers who have at least 18 months experience at the site of investigation (ophthalmology department of Intercommunal hospital-Créteil) or photographers who have already been certified for fundus and angiography photographs in another study will be authorized to perform photographs.

***Protocol for Study Photographs***

The following procedures establish common standards for the study’s photographic work and will ease efficient reading of digital images. Photographers must send digital images according to these guidelines. Photographers are to send digital images to the LERISS by daily e.mail or by floppy or CD-Rom monthly. Each file should be named correctly.

LERISS staff designed these procedures to allow the study site to use the same file format (file format tiff or may be jpeg format no compression) all along the study. The LERISS Director will approve changes to the protocol only to improve quality or maintain study standards.

LERISS staff recognizes that the nature of Age-related macular degeneration may affect image quality. Photographers’ technical skill and persistence are crucial to securing the images necessary to ensure a valid, valuable study.

***Fundus Photography Procedures***

For each patient, study photographers will take red-free fundus photographs at enrolment and follow-up visits at 6 months and on years 1, 2 and 3 (and at an efficacy end point). The quantification of drusen will be based on these digital images.

The study’s preferred camera is a digital angiograph with 50-degree angle fundus CCD camera (Topcon camera, Topcon 50IA, Tokyo, Japan). In each case, photographers should:

- Use the same CCD camera for all the examinations of a same patient.
- Use image file format : tiff or may be jpeg no compression .

Photographers will take fundus red-free images of the study eye at each patient examination. This study’s protocol does not require stereo. Photographers are to take the macula.

The required field is described below and illustrated on the following page:

Field 2 Monocular image of Macula : macula centered.

***Identifying Photographic Materials***

Fundus red-free photographs will be printed out and archived in the patient’s hospital file. The following data must be written on the printed out photographs: patient number (randomisation number eg: 001) and patient first 3 letters of name and surname, Visit number (from V1 to V5), Date of photography (dd/mm/yy) and Photographer ID, all clearly.

Image files will be duplicate. The photographer will keep the original. Image files will be named correctly as the printed out photographs.

The following data must be mentioned : patient number (randomisation number eg: 001) and patient 3 first letters of name and surname, Visit number (from V1 to V5), Date of photography (dd/mm/yy) and Photographer ID, all clearly.

Each day, after performing the exams, the data will be collected by the study nurse.

***Shipping Photographic Materials***

Photographers should email image files directly to LERISS at the following address.

If it is not possible, they may send these image files by floppy or CD-Rom. In this last case, photographers or their staff should fax a copy of the photography log to the LERISS so LERISS staff are advised of the shipping date.

e.mail:

[bunel@univ-paris12.fr](mailto:bunel@univ-paris12.fr)

details:

LERISS (Laboratoire d’Etude et de Recherche en Instrumentation, Signaux et Systèmes)–

**Mr BUNEL** - 69 avenue du Général de Gaulle – 94 010 Créteil - **Telephone** 01 45 17 14 74.

Right Eye

3

**2** x

**1**

**4**

**5**

Left Eye

**1**

X **2**

**3**

**4**

**5**

***Automatic quantification of drusen***

Automatic quantification of drusen is performed using a image enhancement and automatic detection and quantification of drusen software (A. Thaibaoui). The used methods were scientifically validated (see references).

For the automatic quantification, the following steps will be used :

- Image enhancement (grey level histogram enhancement).
- Automatic detection of drusen with sector localization.
- Automatic quantification and classification of drusen (number, size, area) per sector and global.

For evaluation of the drusen evolution, the 2 following steps will be used:

- Geometrical overlaying of images.
- Automatic measurement of variation of drusen number and of area for each drusen.

***References***:

Raji A, Thaibaoui A, Petit E, Bunel P, Mimoun G. A gray-level transformation-based method for image enhancement. Pattern Recognition Letters 1998;19:1207-1212.

Thaibaoui A, Raji A, Bunel P, Petit E. Une nouvelle approche de segmentation des drusen sur des images d’angiographie rétinienne. ITBM 2002 (May) ;23 :235-242.

Thaibaoui A, Bunel P, Mimoun G, Soubrane G. Detection of drusen in retinal fluorescein angiographic images using image analysis. World Congress in Medical Physics & Biomedical Engineering, Nice, 14-19 September 1997; 728.

Thaibaoui A, Raji A, Bunel P. A fuzzy logic approach to drusen detection in retinal angiographic images. IEEE Int Symp on Tech and Soc ISTAS, Rome, 6-8 September 2000; 748-751.

### I. C DIGITAL FLUORESCEIN AND INDOCYANINE GREEN (ICG) ANGIOGRAPHY

Photographers perform fluorescein angiograms (FA) to document and evaluate changes in Macula (CNV and drusen) that are related to age-related macular degeneration. Photographers will conduct fluorescein angiograms at enrolment and at years 1, 2 and 3 (or at CNV occurrence in between) and ICG angiograms in case of suspicion of CNV occurence. FA will be performed with a digital camera (Topcon 50IA, Tokyo, Japan). ICG will be performed using a scanning laser ophthalmoscope (Heidelberg Engineering, Heidelberg, Germany).

Photographers must NOT perform fluorescein angiography on any patient with a history of an allergic reaction to fluorescein injections or ICG angiography on any patient with a history of an allergic reaction to ICG injections.

Photographers should use the same camera to perform all photographs for a same patient all along the study. This is necessary to be able to compare images between the different visits.

***Fluorescein Injection***

- Inject 5 cc 10% Sodium Fluorescein rapidly (minimum of 1 cc per second) into the ante-cubital vein or a large hand vein with a 21-gauge butterfly needle.

***ICG Injection***

- Inject 5 cc 5% glucose solution of ICG as described for fluorescein.

***Autofluorescence and Fluorescein Sequence***

- Take a **red free** filter photograph of the study eye, as well as a photograph of the non study eye. Be sure to be focus on the retinal vessels on the posterior pole.
- For **Autofluorescence**, take images using the blue light, the barrier, and increasing the intensity of the flash.
- Start timer at the very beginning of the **fluorescein injection.**
- Take early phase photographs of the study eye. Begin photographing about 10 seconds after the start of the injection.
- On the study eye, a minimal number of pictures are required, as follows:
  - 2 pictures on the arterial phase
  - 2 pictures on the early venous phase
  - 2 pictures at the complete filling of retinal veins
  - 3 pictures between 0’40 and 1’00
  - 3 pictures between 1’30 and 2’00
  - 3 pictures between 3’00 and 3’30
  - 3 pictures between 4’30 and 5’00.
- On the fellow eye (affected with choroidal new-vessels) the timing of FA acquisition will be performed as usually required for diagnosis and evaluation of the evolution of the choroidal new-vessels (eg, arterial phase, early venous phase, venous phase, intermediate phase at 1’30 – 2’00 and late phase at 4’30 – 5’00.

***ICG Sequence***

- Take a infra-red filter photograph of the study eye, as well as a photograph of the non study eye. Be sure to be focus on the retinal vessels on the posterior pole.
- Start timer at the very beginning of the injection.

Concerning the study eye:

- Take early phase photographs of the study eye. Begin photographing about 5 seconds after the start of the injection.
- Continue a rapid series of photographs from the beginning of filling of the arterial choroidal vessels until the end of the filling of the venous retinal vessels.
- Following photographs will be performed approximately 5, 15 and 30 minutes after the start of the injection.

For the fellow eye, the procedure will be performed as usually required for evaluation of the evolution of CNV, depending on the stage of the disease: arterial choroidal phase to retinal venous phase, and frames at 5, 15 and 30 minutes after the start of the injection.

***Identifying Photographic Materials***

The printed out photographies that will be archived in the patient file and the image files saved on floppy or CD-Rom must be clearly identified.

The following data must be mentioned patient ID and study number, Visit number, Date of photography and Photographer ID all clearly noted.

### I. D BIOLOGICAL ANALYSES

They will consist in a 12 hour fasting plasma lipid profile and in a measurement of total serum and red blood cell membrane (RBCM) fatty acids.

Blood samples must be taken as follows:

- - 5 ml in a dry tube for plasma lipid profile;
  - 5 ml in a dry tube (with clotting gel) for serum fatty acids
  - 5 ml in a lithium heparin coated tube for RBCM fatty acids.

Blood samples must be transported at ambient temperature and delivered to the laboratory within 5 hours of taking.

The fasting lipid profile will include measurements of total cholesterol (TC) and triglycerides (TG) by enzymatic methods (cholesterol esterase / cholesterol oxydase for TC and lipases for TG) (Roche Diagnostics).

Plasma HDL cholesterol (HDL-C) and LDL cholesterol (LDL-C) will be measured by a direct method: enzymatic staining of plasma lipoproteins separated by electrophoresis (SEBIA).

References :

Rifai N, Warnick G, Dominiczak M. Handbook of lipoprotein testing. AACC Press – 1997.

Benlian P. et al. Comparison of a new method for the direct and simultaneous assessment of LDL and HDL cholesterol with ultracentrifugation and usual methods. Clinical Chemistry 2000; 46: 493-505.

Fatty acids in serum and in RBCM will be measured by gas chromatography after extraction with organic solvents. The extracted free fatty acids will be methylated for 5 minutes with freshly prepared diazomethane; fatty acid methyl esters will be gas chromatographed on a polar capillary column coated with Supelcowax-10-bound phase (i.d. 0;32 mm, length 30 m, film thickness 0.25 μm; Supelco, Bellafonte, PA) fitted in a Hewlett-Packard (Palo Alto, CA) gas chromatograph.

Reference :

Klapisz E, Masliah J, Béréziat G, Wolf C, Koumanov K. Sphingolipids and cholesterol modulate membrane susceptibility to cytosolic phospholipase A2. Journal of lipid research 2000; 41 : 1680-1688.

### I. E APO E GENOTYPING

Blood samples must be taken as follows:

- - 10 ml in a EDTA tube.

Blood samples must be transported at ambient temperature and delivered to the laboratory within 5 hours of taking.

The three genotypes corresponding to E2, E3 and E4 alleles (e.g : Cys 112 / Cys 158 for E2; Cys 112 / Arg 158 for E3; Arg 112 / Arg 158 for E4) will be analysed by a primer extension assay using fluorescent dideoxynucleotides.

Genomic DNA will be extracted from circulating white blood cells (WBC) using a sephadex G50 column after proteinase K digestion (QUIAGEN® ). Polymerase chain reaction (PCR) amplification of exon 4 will be performed followed by a multiplex primer extension assay. Fluorescent products will be analysed on a capillary DNA sequencer (ABI 3100; Applied Biosystems).

Reference :

Wang W, Kham S, Yeo G-H, Quah T-C, Chong S. Multiplex minisequencing screen for common southeast asian and Indian β-thalassemia mutations. Clinical Chemistry 2003; 49, 2 : 209-218.

### I. F LENS OPACITY CLASSIFICATION SYSTEM (LOCSII)

Using the slit lamp beam through the dilated pupil, lens opacity will be graded by the investigator using the LOCS II system. The standard photos for grading will be supplied.

Reference :

Chylack L et al. Lens Opacities Classification System II (LOCS II). Arch Ophthalmol 1989; 107 : 995-997.

### I. G MULTIFOCAL ERG.

Multifocal electroretinography is a new technique that allows analysis of local retinal function. The multifocal ERG is a technique for assessing the local ERG from different regions of the posterior retina.

Electrical responses from the eye are recorded with a corneal electrode just as in conventional ERG recording, but the special nature of stimulus and analysis produces a topographic map of ERG responses.

The retina is stimulated with a computer monitor or other device that generates a pattern of elements (typically hexagons).

The patient should be seated comfortably in front of a 20 inches color screen.

The viewing distance will vary with screen size, in order to control the area (visual angle) of retina being stimulated.

The pupils should be dilated.

The different electrodes must be placed as follows :

- Positive on the study eye’s anesthesied cornea,

- Negative, sticked to the temporal side of the eye

- Neutral sticked to the forehead.

To obtain the image clarity, a +3,00 dp correction should be placed in front of the study eye. The other eye should be hidden.

The study eye will be directed to fixate the centre of the cross projected on the screen.

The eye must be kept light-adapted at the room illumination until the experiment begins and room lights must be left on during the examination. The patient should not be exposed to bright sun before this examination. No fundus photography must be performed prior to this examination.

Total time of examination is typically about 8 minutes for a stimulus of 103 elements .

# APPENDIX II. PATIENT INFORMATION AND CONSENT FORM

# APPENDIX III. ADMINISTRATIVE DOCUMENTS

APPROVAL OF CCPPRB

INSURANCE CERTIFICATE

# APPENDIX IV. JUSTIFICATION OF LAWFUL REGULARITY OF THE STUDIED PRODUCT
